# Enhancement of germination and yield of cotton through optical seed priming: Lab. and diverse environment studies

**DOI:** 10.1371/journal.pone.0288255

**Published:** 2023-07-20

**Authors:** Babar Manzoor Atta, Muhammad Saleem, Saifullah Abro, Muhammad Rizwan, Ghulam Sarwar, Amjad Farooq

**Affiliations:** 1 Agri. & Biophotonics Division, National Institute of Lasers and Optronics College, Pakistan Institute of Engineering and Applied Sciences, Nilore, Islamabad, Pakistan; 2 Plant Breeding and Genetics Division, Nuclear Institute of Agriculture (NIA), Tando Jam, Sindh, Pakistan; 3 Cotton Research Station, Ayub Agricultural Research Institute, Faisalabad, Pakistan; PMAS-AAUR: PMAS-Arid Agriculture University Rawalpindi, PAKISTAN

## Abstract

The current study demonstrates the practical application of optical seed priming technology to improve cotton seed germination, plant growth, crop yield, and fiber quality. The hypothesis of this study is that seed irradiation with different colors of light can improve germination and cotton productivity in different environments. In the priming of cotton seeds, a wider range of the light spectrum was used, ranging from ultraviolet (UV) to red wavelengths. Various light sources such as blue LED, red LED, diode laser, UV-B, and UV-C were studied, along with different exposure times and energy densities. The exposure time ranged from 1.0 to 36.0 minutes, while the energy density doses varied from 88 to 7550 mJ cm^-2^, depending on the light source. In laboratory conditions, the investigation on the impact of optical seed priming on germination showed a maximum improvement of up to 180% compared to the control group. Among the different light sources and energy densities, blue LED light was found to be the most effective for enhancing cotton seed germination across different varieties. To validate the findings from the laboratory, large-scale field trials were conducted in two different environments in Pakistan, namely Tandojam and Faisalabad. The field trials demonstrated significant improvements in germination and yield, with increases of up to 37% and 74% over the control group, respectively. Once again, blue LED light emerged as the best light source for optical seed priming at the farm level. These field trials provided encouraging results, indicating the potential of the eco-friendly optical seed priming technique. The study suggests that optical seed priming can be a commercially viable technology for improving cotton seed germination, plant growth, crop yield, and fiber quality. By utilizing this technique, growers and researchers in developing countries can address the challenge of poor cotton germination and potentially enhance their agricultural productivity.

## Introduction

Seed has inherent potential for crop performance and is believed to be the essential input in agriculture [1]. Good quality seed exhibit high vigor, leading to plant uniformity, increased crop productivity (up to 30%), and the assurance of marketing and food security [2]. However, poor cotton seed germination remains a significant challenge for farmers and researchers in developing countries, where achieving germination rates of only 40–45% is common, compared to over 95% in some advanced countries. If germination rates can be enhanced to above 80%, farmers can use only 3 kg of cotton seed per acre instead of the current requirement of 6–8 kg. Higher seed germination and rapid seedling establishment are prerequisites for profitable production since these stages are the most vulnerable in the crop life cycle [3].

Cotton (*Gossypium hirsutum* L.) is well-known in Pakistan for its 5F (Food, Fiber, Feed, Fuel, and Fodder) contributions. It is the second most important cash crop in the country and significantly contributes to the national economy. However, the yield and cultivated area of cotton are declining [4] due to various reasons, including issues with seed germination [5]. Therefore, it is crucial to address this problem and find a sustainable solution. Several seed enhancement techniques are being used to improve germination and establish healthy crops in changing environments [2, 6]. One such technique is the use of laser/light irradiation, which has been reported in the literature for seed invigoration in various agronomic and horticultural crops.

Different colors of light stimulate different aspects of seed germination, seedling development, plant health, and growth [7], thereby increasing crop yield and quality [8, 9]. Light spectrum strongly influences seed germination, seedling development, and plant physiology. The application of laser/light bio-stimulation mechanisms has practical uses in agriculture, particularly for farmers and the seed sector. Hernandez et al. [7] described the basis of the stimulation mechanism, stating that seeds absorb light through photoreceptors, which convert light energy into chemical energy. This energy activation triggers physiological and biochemical processes, initiating various biological reactions. The authors reviewed three main types of photoreceptors in plants: i) phytochromes, which detect red and near-infrared light (600–750 nm) and mediate seed germination, seedling development, and flowering; ii) blue/ultraviolet (UV-A) absorbing cryptochromes, which are blue light (320 to 500 nm) photoreceptors involved in photo-morphogenesis and growth responses; and iii) phototropins, which are blue-light receptors controlling light-induced responses that optimize plant photosynthetic efficiency, including phototropism, chloroplast movements, leaf expansion, and stomatal opening, leading to efficient light energy capture, reduced photo-damage, and increased CO_2_ uptake [10, 11].

Among physical methods, priming with light, including ultraviolet (UV-A, UV-B, UV-C) rays, is a useful and eco-friendly seed treatment approach to improve crop yield and quality under stressful environments [8, 9]. Treating seeds or seedlings with lower doses of UV-B and UV-C promotes the production/activity of various metabolites, enzymatic and non-enzymatic antioxidants, enhancing plant tolerance to abiotic stresses [9]. The use of light-emitting diodes (LEDs) for seed priming is a cost-effective and efficient method of irradiation [12]. The use of specific compositions of LED lights in food production and preservation has shown increased yields, as well as improved nutritive content and quality in horticultural and agricultural produce [13].

Although the beneficial effects of laser/light have been reported in various crops, there is no significant practical work on cotton crop in the literature. The hypothesis of this study is that seed irradiation with different colors of light can improve germination and cotton productivity in different environments. While most investigations on the effects of seed priming technology using different light colors have been conducted under in vitro conditions, there is a lack of research on the effects under field conditions. This study represents the first report on the priming effect of different light colors on cotton seed germination rate under in vitro conditions and the effects of optimized doses on plant growth and yield under ex vitro conditions.

The findings of this research will benefit researchers, the seed industry, and cotton farmers in improving cotton seed quality and contributing to sustainable agriculture. The main objectives of this study are: i) to optimize a wider range of light spectrum for enhancing seed quality in cotton, ii) to investigate the impact of optical seed priming technology on cotton germination under controlled conditions, iii) to evaluate the impact of optical seed priming technology on cotton performance in two diverse agro-ecological conditions, and iv) to propose a low-cost optical technology for cotton seed enhancement.

## Materials and methods

### Seed material

The testing material was acquired from different sources including: Central Cotton Research Institute (CCRI), Multan; Nuclear Institute of Agriculture (NIA), Tandojam and Cotton Research Station (CRS), Faisalabad comprised of the following cotton varieties: Cyto-124 (Bold seed; delinted), NIA-Noori (Bold seed and fuzzy seed), Sadori (Bold seed and fuzzy seed), FH-490 (Bold seed and fuzzy seed), and FH-492 (Bold seed and fuzzy seed). Fuzzy seeds are characterized by the presence of lint on the seed surface. However, in commercial settings, lint is typically removed from bold seeds using a treatment process involving commercial-grade sulphuric acid.

### Light sources and light energy densities

Seed priming work was initiated and performed at National Institute of Lasers and Optronics (NILOP), Nilore, Islamabad, Pakistan. In the priming of cotton seeds, wider range of the light spectrum was used starting from ultra violet (254 nm) to the red (645 nm) wavelengths. Different light sources i.e. blue LED, red LED, diode laser, UV-B & UV-C and exposure times/energy densities have been studied extensively. The details of the laser/light sources used in the studies are given in the Table 1.

**Table 1:** Detail of the light sources and the energy densities used in the optical seed priming studies.

Laser/Light	Color	Wavelength (nm)	Exposure time (minutes)	Energy density (mJ cm^-2^)
Blue LED	Blue	455 nm	1.0–26.0	290–7550
Red LED	Red	645 nm	1.0–36.0	199–7152
Diode Laser	Red	635 nm	1.0–19.0	306–5807
UV-B	Purple/Ultra Violet	UV-B band (280–315 nm)	1.0–17.0	234–3986
UV-C	Purple/Ultra Violet	UV-C band (254 nm; 100–280 nm)	1.0–25.0	88–2198

Energy density is the irradiation dose (s) used for different lights & laser diode and calculated based on the power, area and the time duration.

### Light irradiation treatment

The healthy seeds of each cotton variety were used as control (non-irradiated) and for irradiation treatment. The seeds were irradiated with different light sources as mentioned in Table 1. In lab. studies, for LED irradiation the distance between the light source and the seed samples was 1.5 inches, whereas in case of diode laser, UV-B and UV-C tube lights the distance was 5 inches with uniform illumination of seeds. Using each light source, the seeds were treated with different exposure times and the total energy densities applied are presented in mJ cm^-2^ (Table 1).

**Fig 1:**
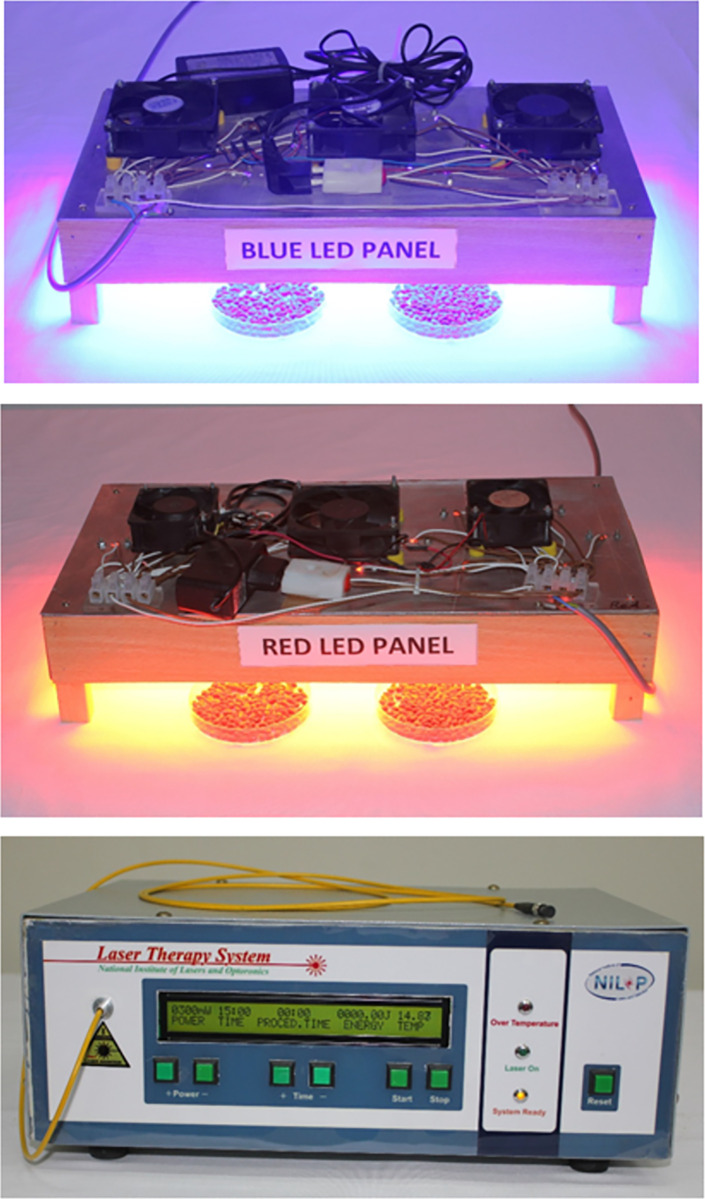
Indigenously developed seed irradiation light systems. Blue LED panel, Red LED panel, and Diode laser system.

To irradiate the larger quantity of seed, a panel each of blue LED and red LED was fabricated in the Lab. of Agri. & Biophotonics Division, NILOP, Islamabad. The panel L x W dimensions are 16" x 9". Each panel comprised of 16 LEDs and 3 cooling fans over the metal plate. The LED to the seed sample distance was 3.5 inches. The seed irradiation light systems developed indigenously at NILOP, Islamabad are presented in Fig 1. After exposure of the seeds to the light, the treated seeds were kept in individual brown paper bags to avoid exposure to any other light source.

### Laboratory studies

#### Experimental layout

The experiments were started by growing the control and the irradiated seeds at NILOP, Islamabad in controlled environment. The seeds of the cotton varieties were raised in plastic containers (15 cm diameter and 7 cm height) placed in the growth room at 32 ± 2°C. Cleaned sand was used as the growing medium. Twenty seeds per replication per treatment were sown at the depth of 2 cm in the sand. Six holes were made under the containers for the drainage of excess water. The experiments were conducted in completely randomized design (CRD) with two replications during October 2020 to May, 2021. Tap water was applied to the growing seeds as required. Sufficient water was applied to give equal opportunity to each seed to germinate by avoiding water stress. The lights of the growth room were kept off to ensure that the treated seeds sown in the containers may not be exposed to any other light source during germination phase.

#### Germination data

The germination data were recorded on 12^th^ day after sowing by counting the germinated seeds for each treatment. The germination (%) and percent increase from control was estimated and presented in the graphs. Standard Error was also calculated and displayed on the control and irradiated treatments in the graphs. The data were compiled and analysed in Excel.

### Field trials

#### Experimentation

The field trials of selected irradiation doses (from the lab. studies) were conducted at two diverse environments in two different districts namely Sindh and Punjab. The following cotton field trials were conducted during 2021 (Table 2).

**Table 2:** The detail of the field trials conducted at two contrasting environments regarding the optical seed priming.

Environment		Location	Trials	Varieties	Light treatments
1	Tandojam	Nuclear Institute of Agriculture (NIA), Sindh	i. Bold seed	NIA Noori, Sadori	Control Blue LED Red LED Diode Laser UV-B UV-C
			ii. Fuzzy seed	NIA Noori, Sadori	
2	Faisalabad	Cotton Research Station (CRS), Punjab	i. Bold seed	FH-490, FH-492	
			ii. Fuzzy seed	FH-490, FH-492	

The selected optimized energy densities from lab. studies for each variety and light source were used in these four cotton field trials. In total four trials were conducted; two at NIA and two at CRS. At NIA, Tandojam the trials were planted on 6^th^ May, 2021 and the trials at CRS, Faisalabad were planted on 21^st^ May, 2021. The non-irradiated seed of the each variety was used as the respective control. The control and the irradiated seeds were planted in randomized complete block design (RCBD) with three replications. The plot size was 18 m^2^ with 4 rows × 6 m long × 0.75 m apart. Plant to plant distance was 1ʹ, whereas row to row distance was kept as 2.5ʹ. Seeds were sown manually with the help of labour. The area covered by each trial was 22 × 36 m. Standard agronomic practices were followed to control weeds.

#### Weather data

Meteorological data for cotton growing season, 2021 regarding temperature, rainfall and humidity were obtained from the nearest weather stations situated at the trial locations as under;

Environment 1, Tandojam (Sindh): Pakistan Meteorological Department, Tandojam

Environment 2, Faisalabad (Punjab): Plant physiology section, Agronomic Research Institute, AARI, Faisalabad.

The weather data have been presented in the S1 and S4 Tables.

#### Soil properties

The soil samples were taken from the cotton trials conducted at Tandojam and Faisalabad during 2021 and analysed for various soil properties. The soil parameters and the type of soil texture have been presented in the S2 and S5 Tables.

#### Agronomic practices

The fertilizer rate and the date/stage of application, irrigation date and application number along with some other agronomic details are mentioned for cotton trials conducted at Tandojam (S3 Table) and Faisalabad (S6 Table). All these practices were carried out for good crop growth with uniform stand.

#### Morphological attributes

The germination data were recorded at the initial growth stage whereas cotton yield (kg ha^-1^) data were collected at maturity up to the last cotton picking stage. Additionally, a set of comprehensive data were generated both for bold seed and fuzzy seed trials conducted at CRS, Faisalabad. The data were recorded on the following parameters; Days to first bud (DFB); Days to first flower (DFF); Plant population per hectare (PPH); Staple length, mm (SL); Micronaire, μg/in (MCN); Fiber strength, g/tex (FS); Ginning out turn, % (GOT); Plant height, cm (PH); No. of monopodial branches per plant (MBP); No. of sympodial branches per plant (SBP); No. of bolls per plant (BP); Nodes to first fruiting branch (NFFB); and Boll weight, gm (BW). The morpho-agronomic data have been presented in the S7 and S8 Tables.

#### Data analysis of field trials

The germination, agro-morphological and yield data of the replicated plots were recorded and analyzed using Statistix 8.1 software. Analysis of variance was conducted for each parameter separately using the randomized complete design in linear models procedure of Statistix 8.1 software. The treatment means were compared by the least significant difference (LSD) test at P = 0.05.

#### Supporting information

The data in the form of tables are presented in the S1 to S15 Tables.

## Results

### Laboratory studies

The seedling studies were conducted in containers in the growth room and results of each light source are discussed here.

### Blue LED (455 nm)

**Fig 2:**
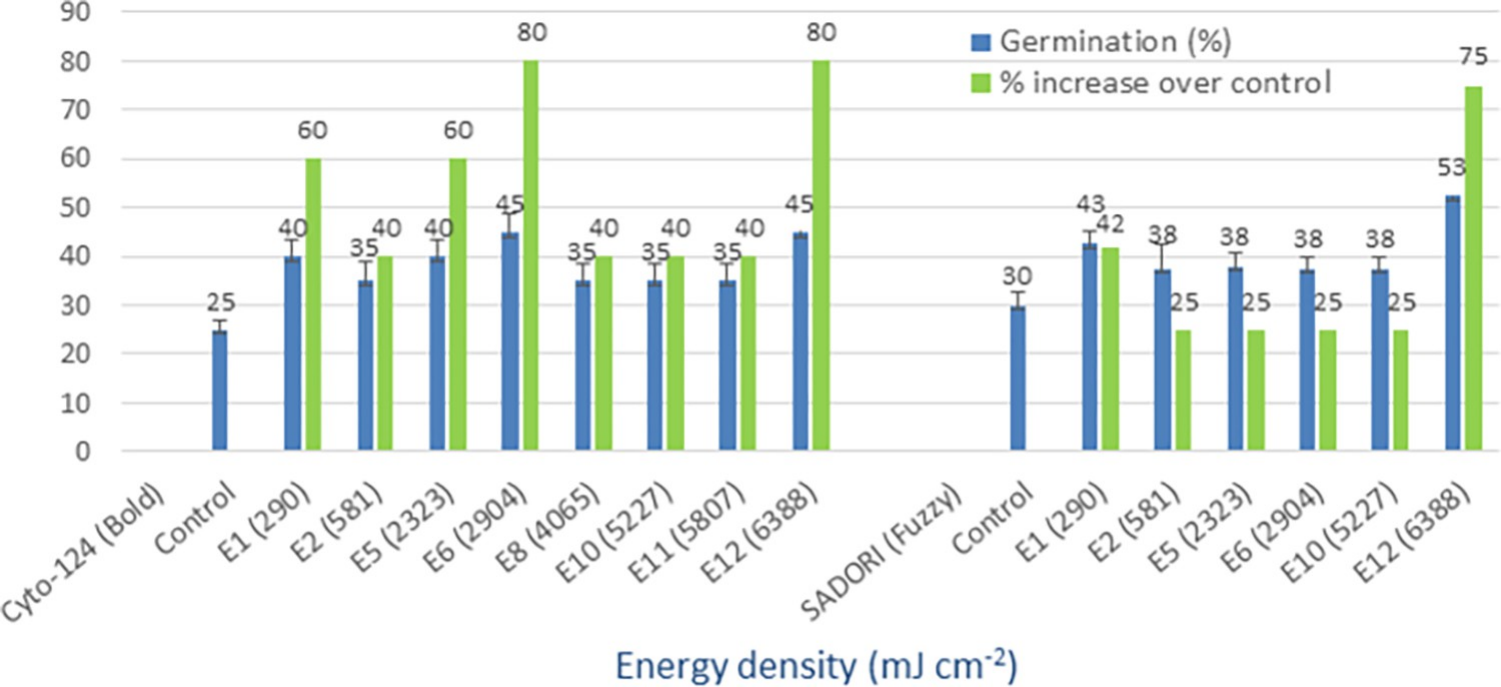
Germination (%) and percent increase in germination over non-irradiated control after irradiation of seeds with blue LED light in controlled environment.

**Fig 3:**
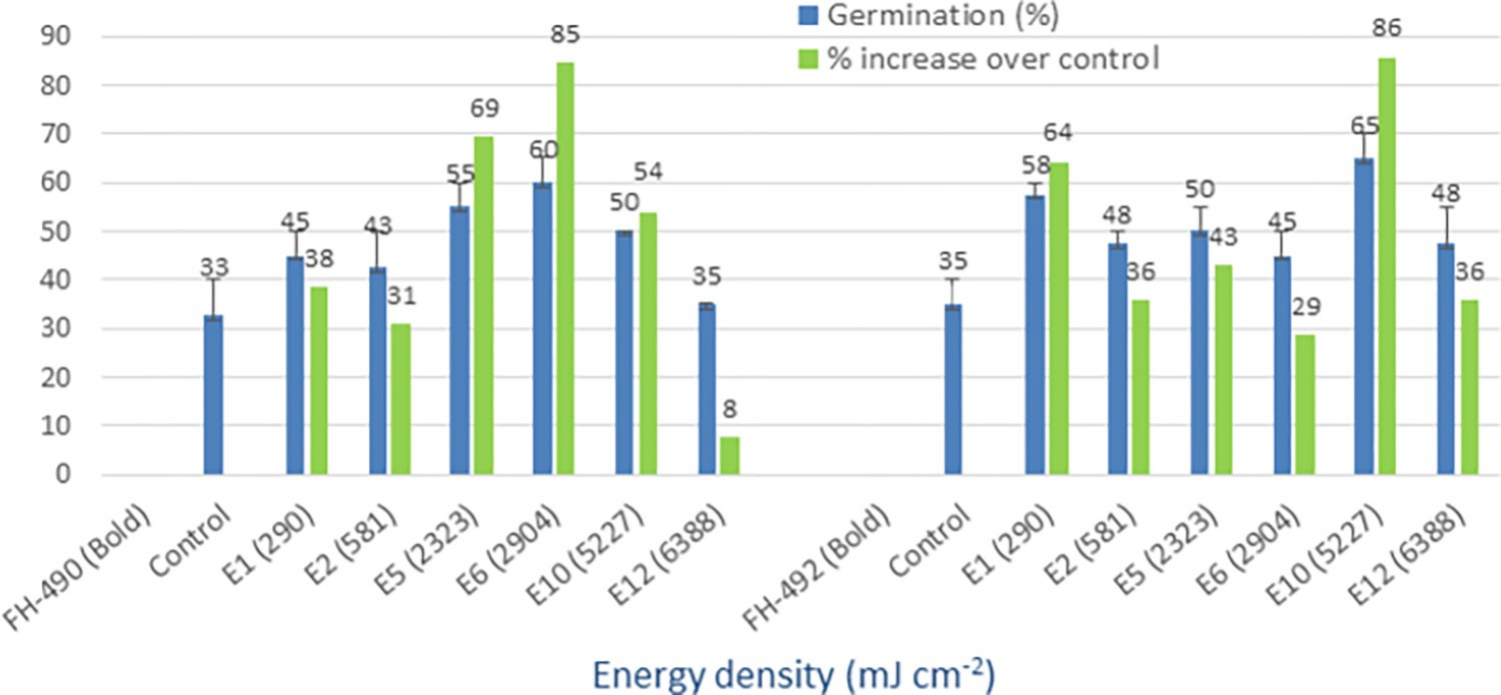
Germination (%) and percent increase in germination over non-irradiated control after irradiation of seeds with blue LED light in controlled environment.

The germination rates of four cotton varieties, namely Cyto-124, Sadori, FH-490, and FH-492, were found to range from 25% to 35% in the control group. However, when these varieties were subjected to irradiation using blue LED light at different energy densities, their germination rates improved significantly, ranging from 45% to 65%. Notably, the irradiated seeds demonstrated a remarkable increase of 75% to 86% in germination compared to the non-irradiated control group (as shown in Figs 2 and 3). The enhanced germination was observed in all cotton varieties/seed types when exposed to specific energy densities measured in millijoules per square centimeter (mJ cm^-2^). The energy densities that yielded the best results were 2904 (E6), 5227 (E10), and 6388 (E12), with duration of the light treatment ranged from 10 to 22 minutes for the seeds. Although there were some varietal differences in response to the energy densities, overall, germination improved across all varieties. The findings indicate that blue LED light is the most effective treatment for optical seed priming of cotton, resulting in improved seed germination.

### Red LED (645 nm)

**Fig 4:**
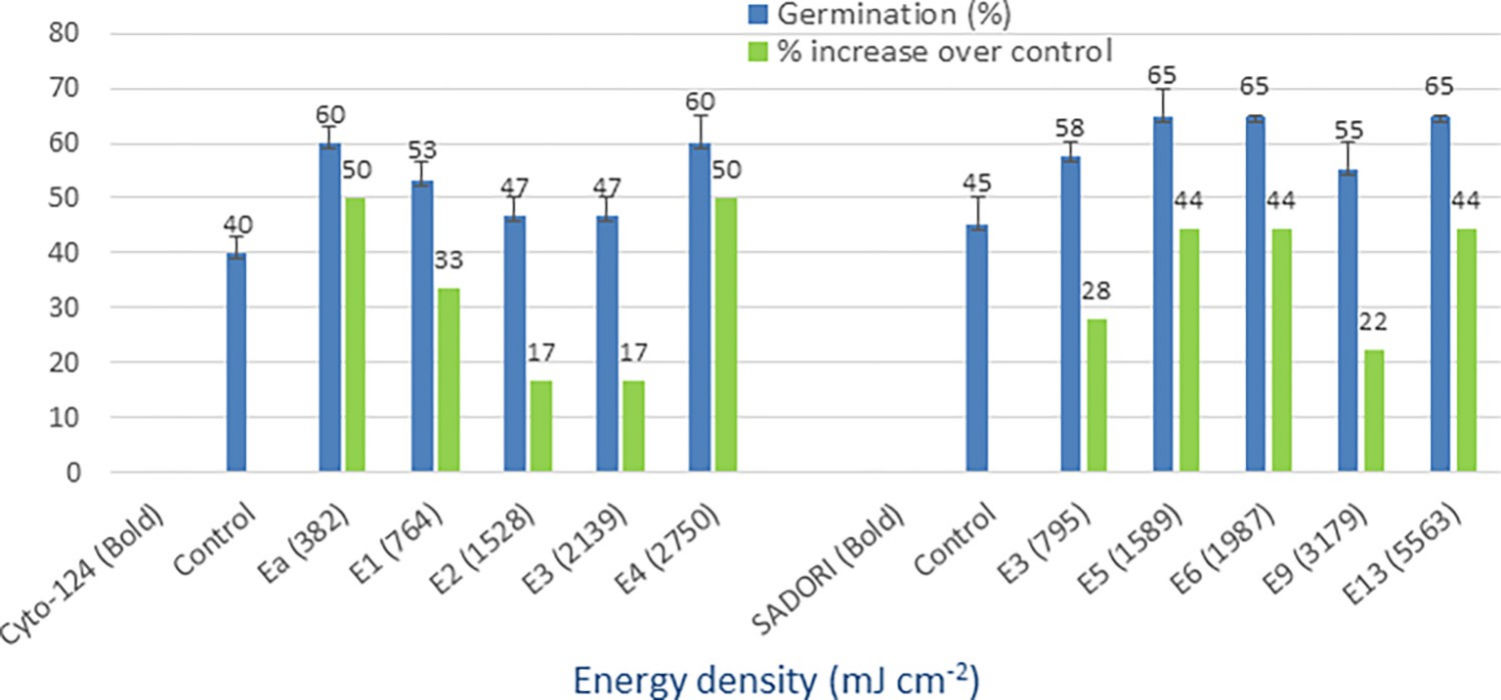
Germination (%) and percent increase in germination over control after seed irradiation with red LED light in controlled environment.

**Fig 5:**
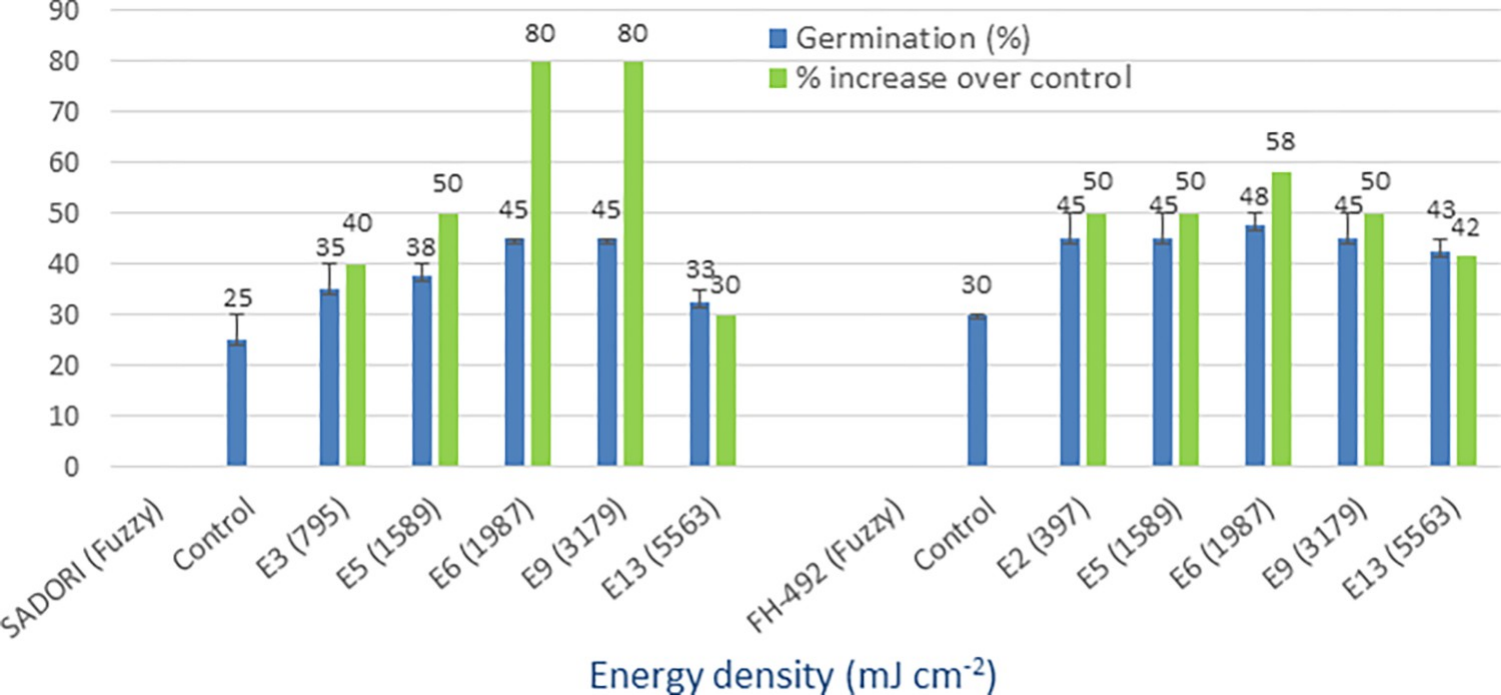
Germination (%) and percent increase in germination over control after seed irradiation with red LED light in controlled environment.

The treated seeds of cotton varieties with red LED light at various energy densities showed a higher mean germination rate (45% to 65%) compared to the non-irradiated controls (25% to 45%). The overall benefit, in terms of percentage increase over the controls, ranged from 44% to 88% among the four tested cotton types (Figs 4 and 5). In most of the varieties, the E6 dose with an energy density of 1987 mJ cm^-2^ and a treatment time of 10 minutes performed better. The minimum percentage increase over the controls achieved was 17% in response to red LED light seed treatment. Therefore, red light irradiation has been found to be promising in all the varieties at different energy densities.

### Diode laser (635 nm)

**Fig 6:**
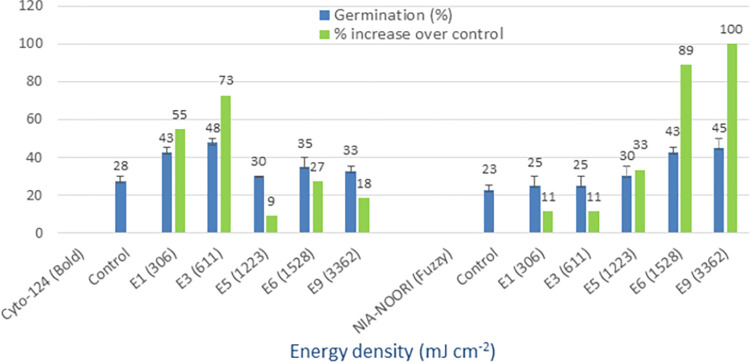
Germination (%) and percent increase in germination over control after seed irradiation with diode laser in controlled environment.

**Fig 7:**
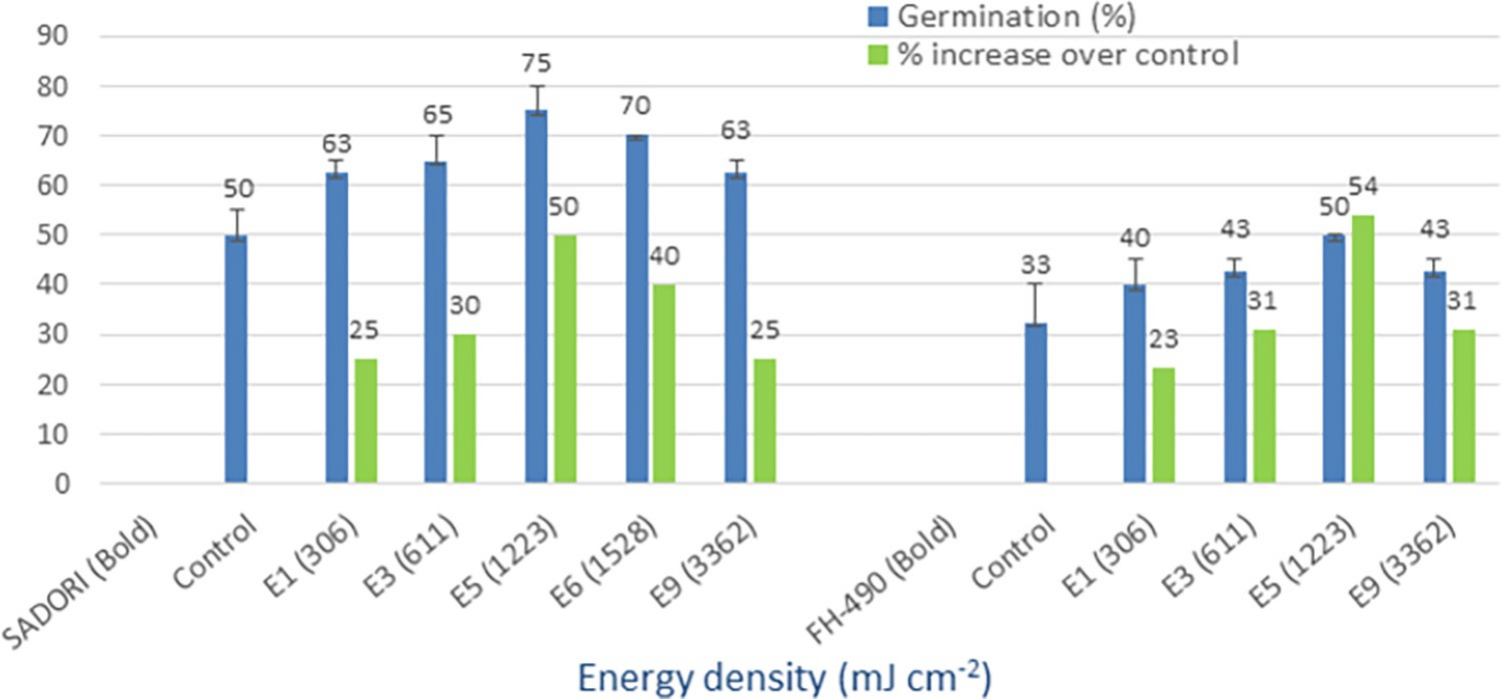
Germination (%) and percent increase in germination over control after seed irradiation with diode laser in controlled environment.

The control treatments of the four tested cotton varieties, namely Cyto-124, NIA-Noori, Sadori, and FH-490, exhibited germination rates of 28%, 23%, 50%, and 33%, respectively (Figs 6 and 7). However, when the cotton seeds were irradiated with a diode laser, the maximum mean germination rates for these varieties were observed to be 48%, 45%, 75%, and 50%, respectively. Additionally, the advantages in terms of percentage increase over the control were 73%, 100%, 50%, and 54% for the Cyto-124, NIA-Noori, Sadori, and FH-490 varieties, respectively. The seed treatment energy densities that yielded the best results were 611 (E3), 3362 (E9), and 1223 (E5) mJ cm^-2^. The tested varieties displayed minimum percentage increases over the control of 9%, 11%, 25%, and 23%, respectively, thus demonstrating the value of this light source for optical seed priming applications.

### UV-B (280–315 nm)

**Fig 8:**
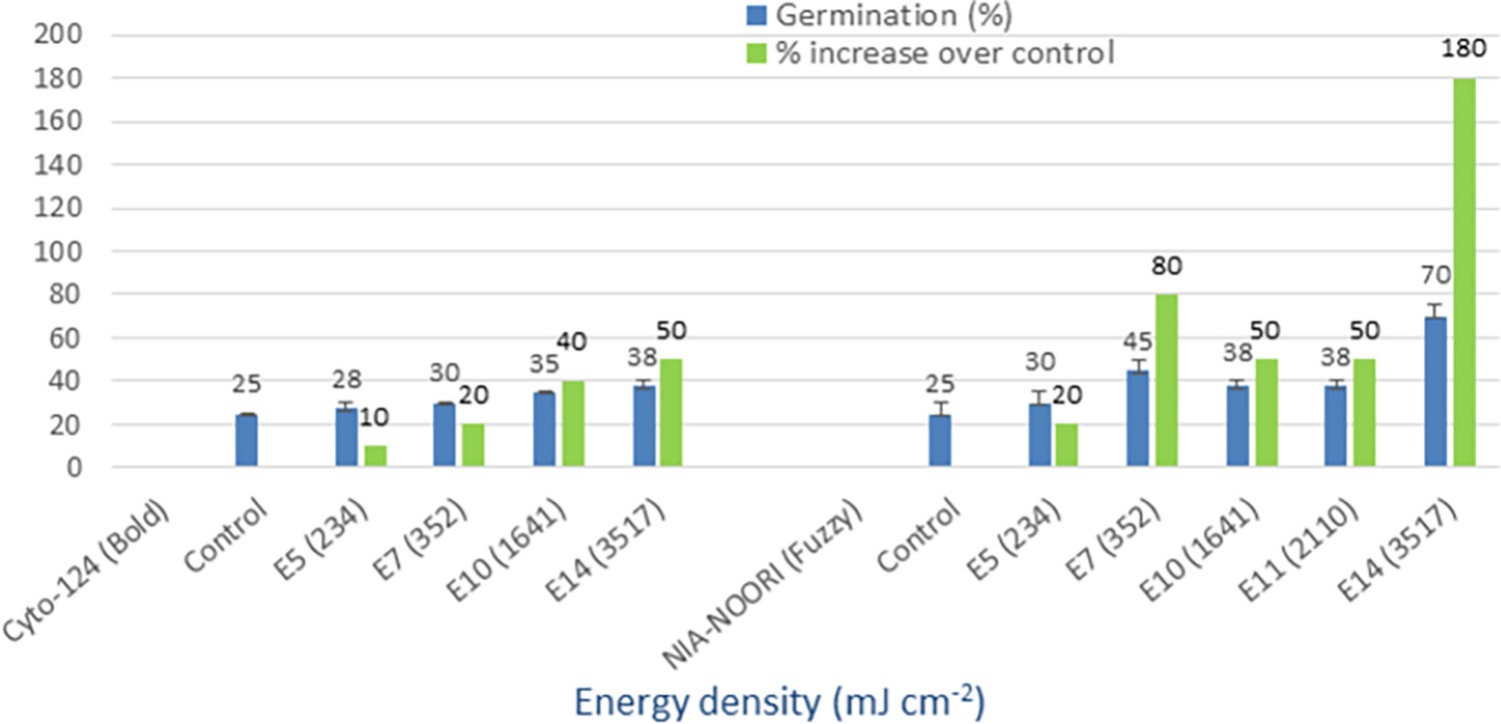
Germination (%) and percent increase in germination over control after seed irradiation with UV-B light in controlled environment.

**Fig 9:**
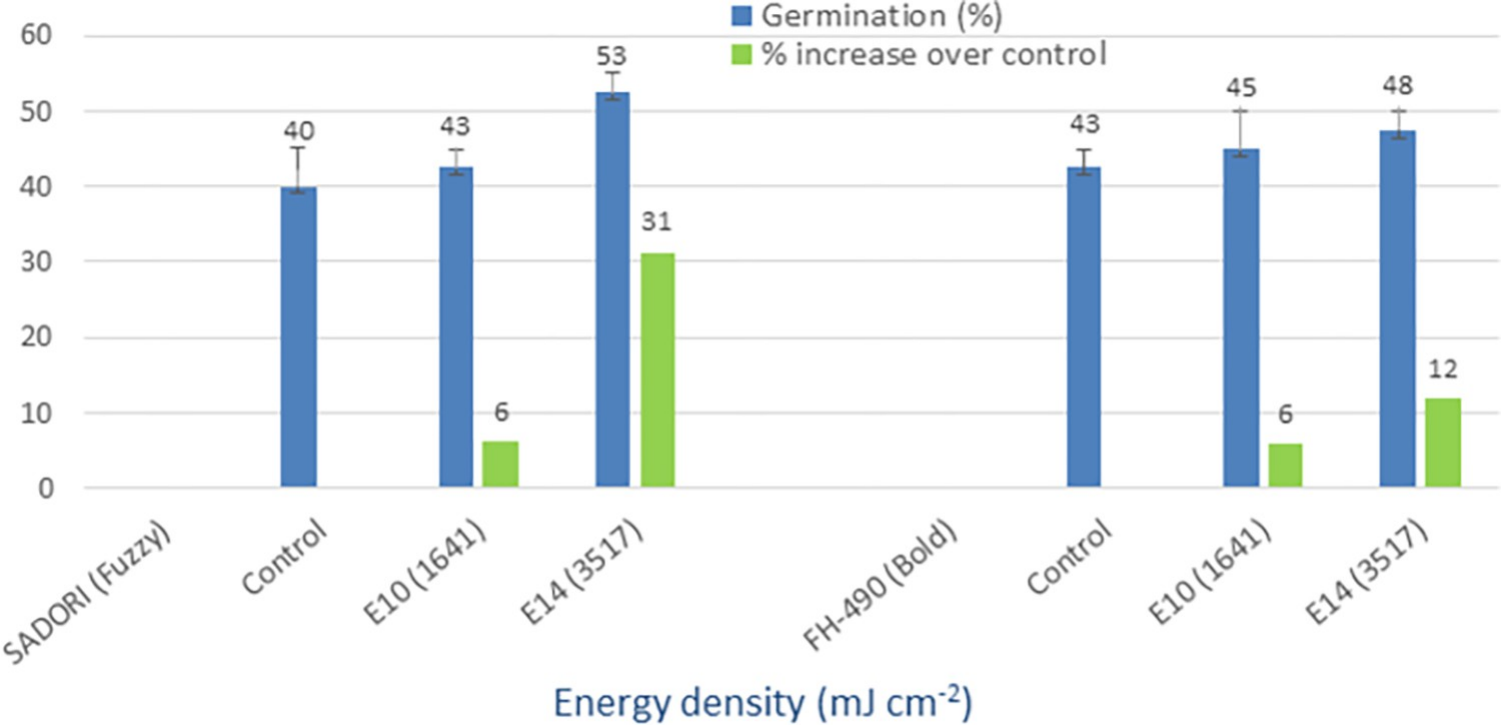
Germination (%) and percent increase in germination over control after seed irradiation with UV-B light in controlled environment.

The mean germination of the control group ranged from 25% to 43%, whereas the irradiation of seeds with UV-B light resulted in improved germination rates of 38% to 70% (Figs 8 and 9). The greatest improvement in germination, expressed as a percentage increase over the control group, was observed in the following varieties: Cyto-124 (50%), NIA-Noori (180%), Sadori (31%), and FH-490 (12%). Among these cultivars, the E14 energy density (3517 mJ cm^-2^) yielded the best results. The minimum percentage increase over the control group ranged from 6% to 20% across these varieties. These findings demonstrate the effectiveness of UV-B irradiation as a method for physically priming cotton seeds.

### UV-C (254 nm)

**Fig 10:**
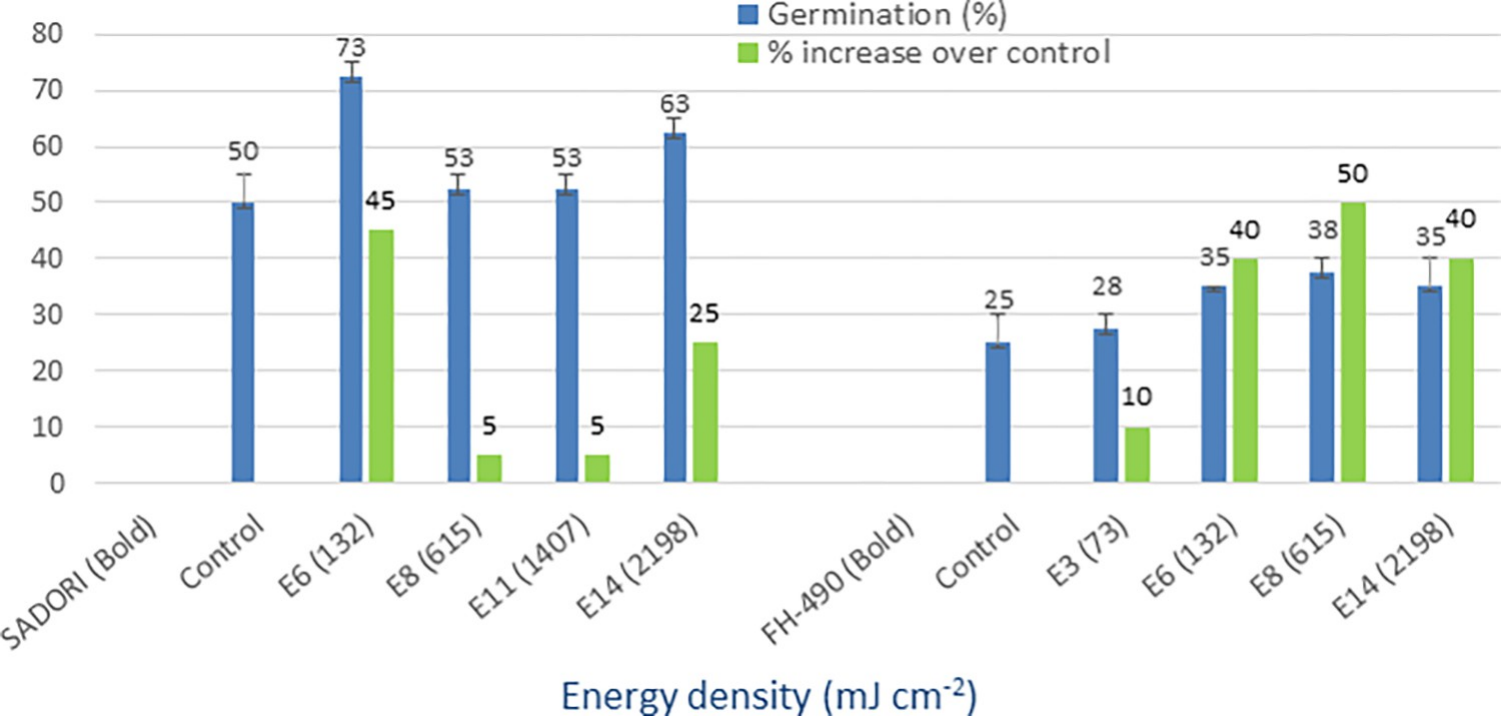
Germination (%) and percent increase in germination over control after seed irradiation with UV-C light in controlled environment.

**Fig 11:**
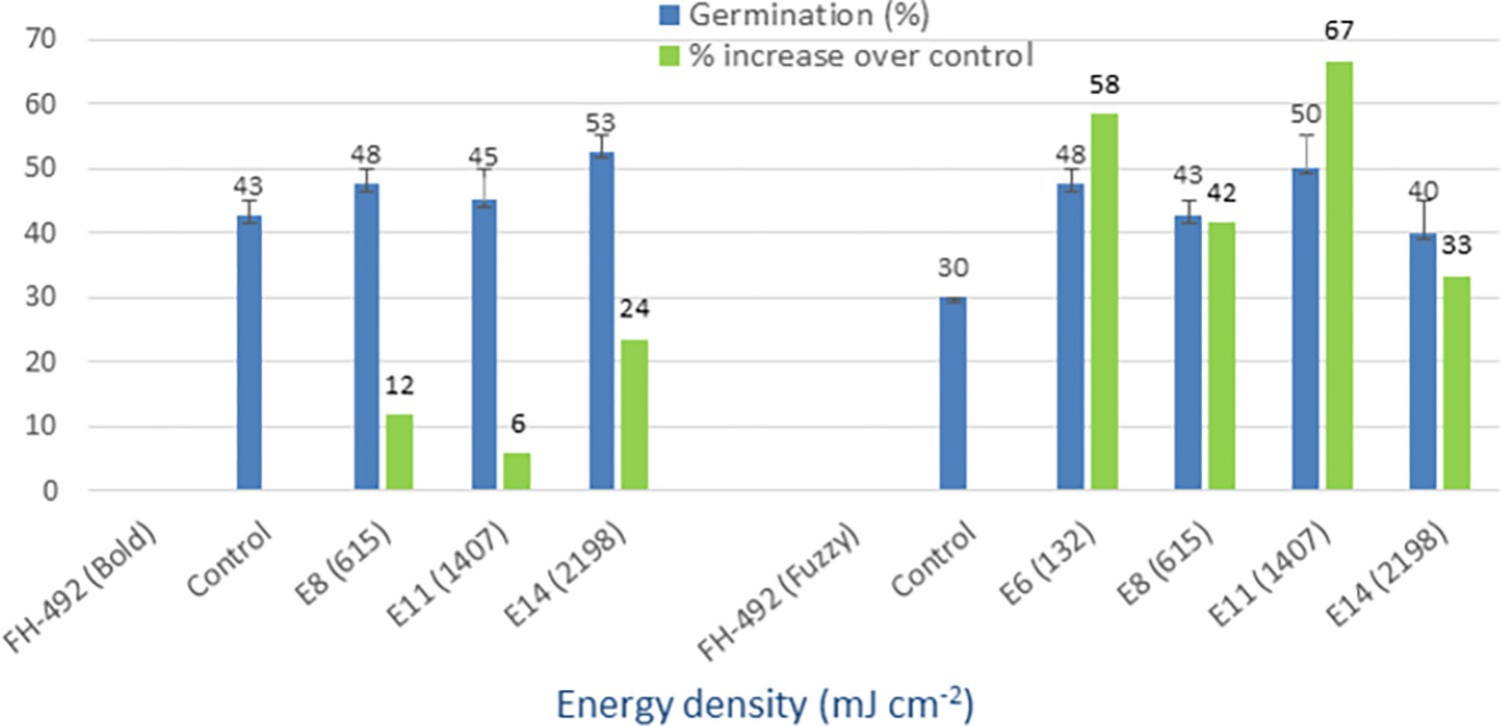
Germination (%) and percent increase in germination over control after seed irradiation with UV-C light in controlled environment.

The irradiation of seeds with UV-C light resulted in significantly higher mean germination percentages compared to the non-irradiated controls. The germination rates for the treated seeds were recorded at 73%, 38%, 53%, and 50%, while the controls exhibited germination rates of 50%, 25%, 43%, and 30% (Figs 10 and 11). Furthermore, the treated varieties displayed a maximum percentage increase in germination over the control group, ranging from 24% to 67%. On the other hand, the minimum increase observed in germination rates for the treated seeds ranged from 5% to 33%. These findings demonstrate the potential of UV-C light treatment as a means to enhance cotton seed germination.

### Field studies

To evaluate the effectiveness of optical seed priming technology in real-world conditions, cotton seeds were exposed to optimized doses of irradiation and then planted in field trials. These trials were conducted in two contrasting environments: the Sindh and Punjab provinces. The first location chosen for the trials was the Nuclear Institute of Agriculture (NIA) in Tandojam, Sindh. The second location was the Cotton Research Station (CRS) in Faisalabad, Punjab. Data on germination (%) and cotton yield (kg ha^-1^) were collected at both locations and presented in figures, along with the estimated percentage increase over the control group.

### Environment 1: Tandojam, Sindh

Tandojam is located at latitude 25°25′37″ N and longitude 68°32′10″ E, with an elevation of 29 meters (95 feet) above sea level [14]. It is situated in the Sindh province of Pakistan. The cotton growing season in 2021 was characterized by dry weather in Tandojam, with a total in-season rainfall of 40 mm (S1 Table). July and September experienced relatively higher rainfall. Due to the low and non-uniform distribution of rainfall, the irrigated areas of Tandojam are well-suited for cotton cultivation. The month of June had the highest mean air and soil temperatures, as well as the highest average sunlight hours. Humidity levels increased initially and then decreased during the season. The soil texture at the trial location was clay loam, with the proportions of sand, silt, and clay being 22.5%, 41.3%, and 36.1%, respectively. The soil had a slightly alkaline pH and a low organic matter content (S2 Table). Fertilizer application involved using Di-ammonium Phosphate (DAP) once at sowing, while Urea was applied in three separate doses (S3 Table). A total of eight irrigations were provided during the season. Additional agronomic practices followed for these trials can be found in S3 Table.

**Fig 12:**
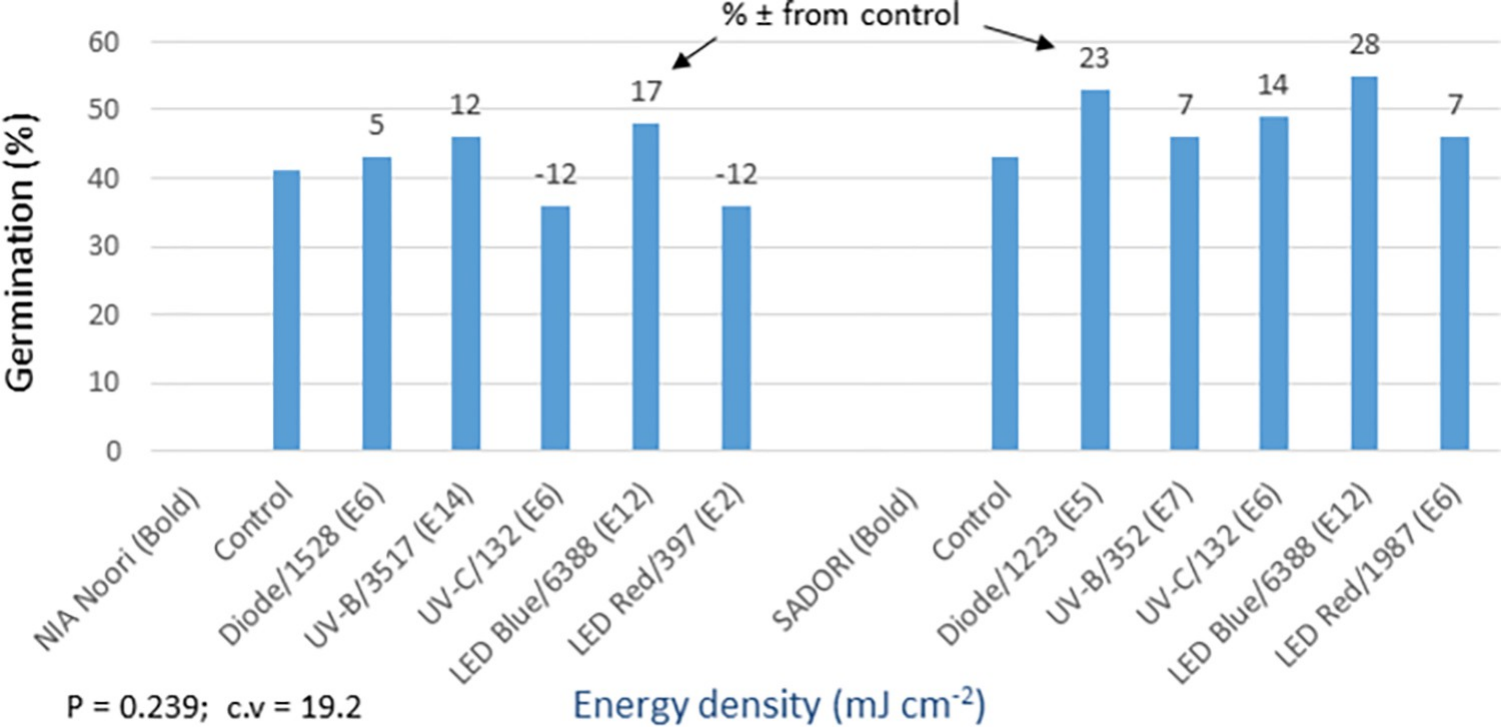
Environment 1 (bold seed trial). Germination (%) and percent increase/decrease (±) in germination over control in field trial conducted at NIA, Tandojam after optical seed priming. LSD, P = 0.239: Non-significant; cv: Coefficient of variation (%).

**Fig 13:**
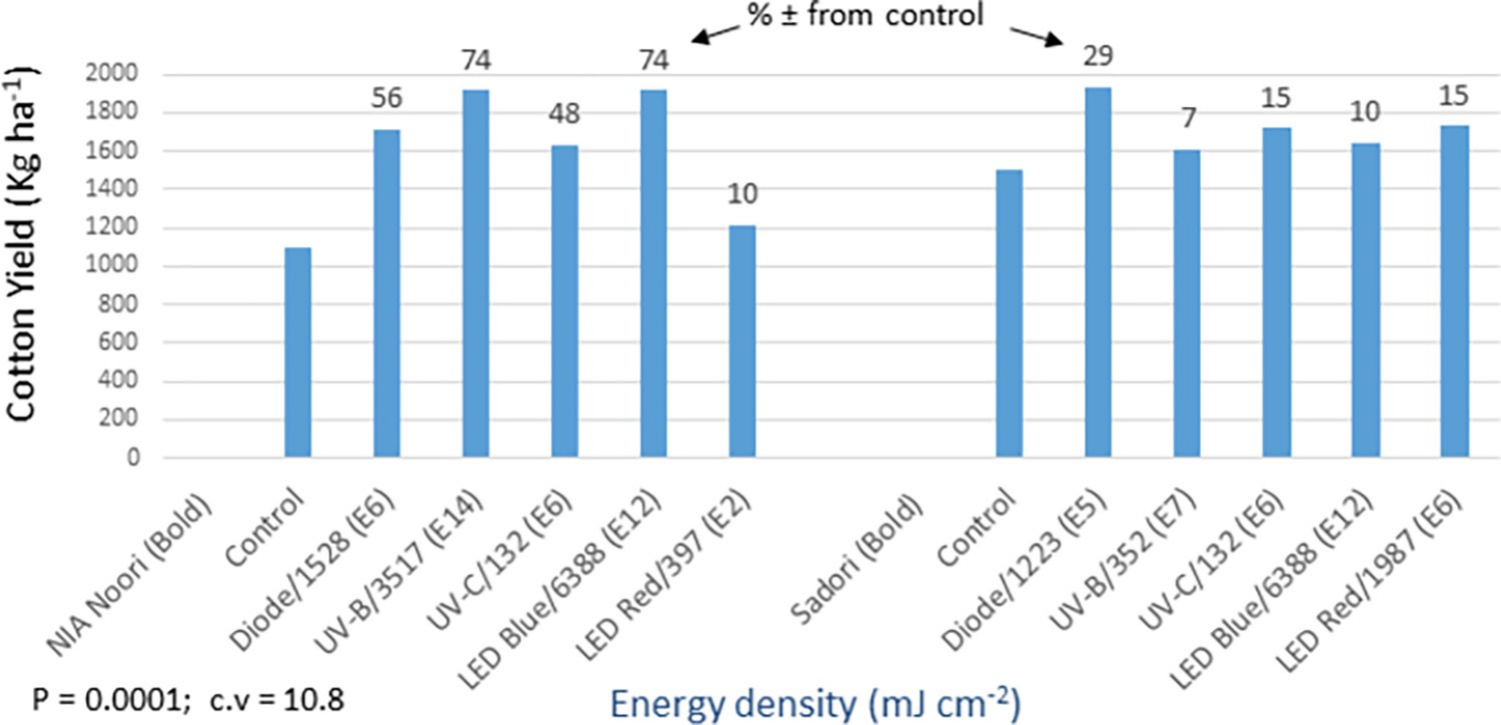
Environment 1 (bold seed trial). Cotton yield (kg ha^-1^) and percent ± in yield over control in field trial conducted at NIA, Tandojam after optical seed priming. LSD, P = 0.0001: Highly significant; cv: Coefficient of variation (%).

**Fig 14:**
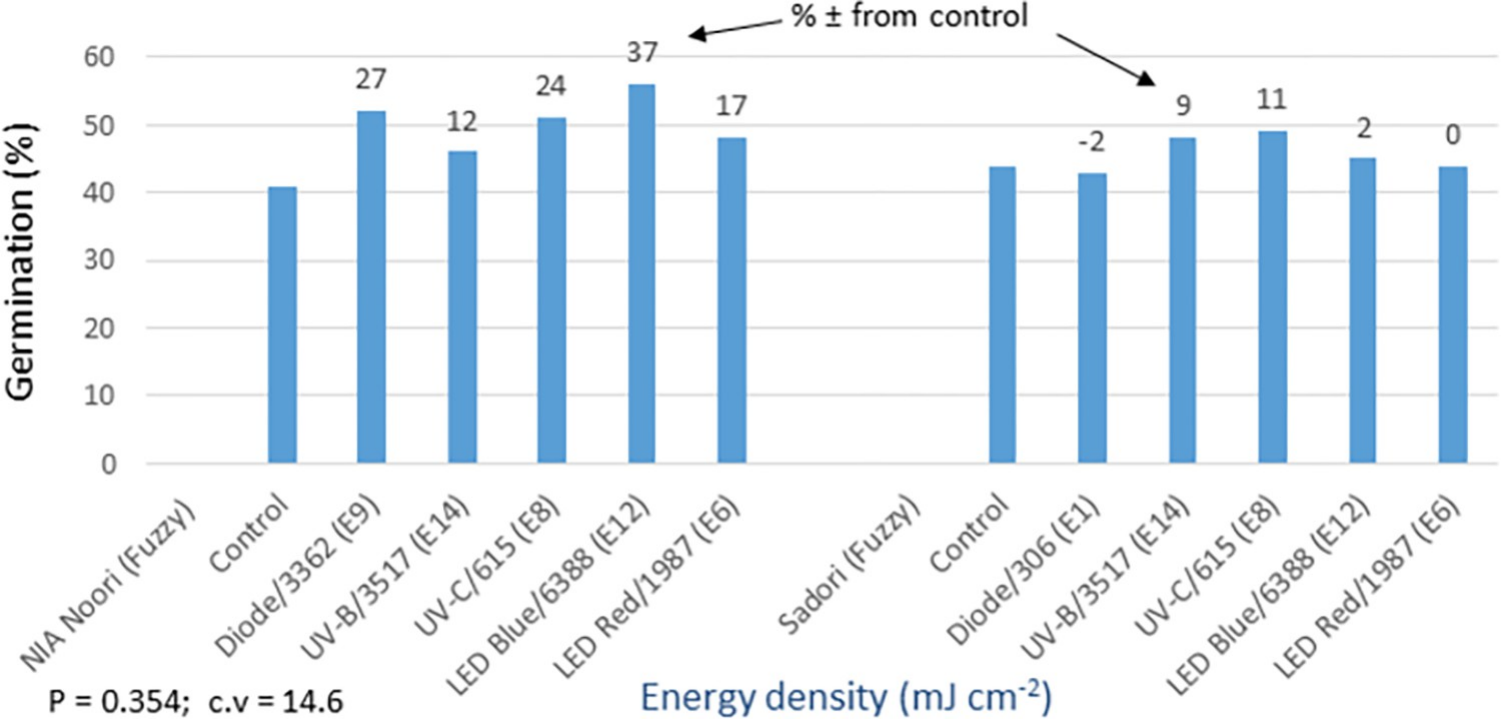
Environment 1 (fuzzy seed trial). Germination (%) and percent ± in germination over control in field trial conducted at NIA, Tandojam after optical seed priming. LSD, P = 0.354: Non-significant; cv: Coefficient of variation (%).

The statistical analysis of germination data for trials involving cotton bold seeds and fuzzy seeds showed non-significant differences between the light treatments (see Figs 12 and 14). Normally, when dealing with cotton seeds that have poor germination rates, it is common practice to sow multiple seeds per hill. However, in these trials, only a single seed was sown per hill. This resulted in lower germination rates and more variation among replicates during the early stages of crop development, which obscured the effects of the light treatments. Furthermore, it was observed that the primed seeds, which were generally weaker, exhibited a tendency to germinate later. In the absence of priming, these seeds might not germinate at all. This led to an improvement in the germination of primed seeds later in the season, as indicated by their significantly different (P = 0.0001) and higher cotton yields at maturity (see Fig 13). In the bold seed trial (see Figs 12 and 13), the following observations were made: i) NIA Noori variety: The blue LED treatment exhibited the highest percentage increase in germination over the control (17%), followed by UV-B (12%) and diode laser (5%). Similarly, these treatments showed the highest yield increase (74% to 56%) compared to the control, ii) Sadori variety: The blue LED treatment displayed an enhanced percentage increase in germination over the control (28%), followed by diode laser (23%) and UV-C (14%). These treatments also showed improved yields ranging from 10% to 29% compared to the control. Overall, all of the light treatments resulted in statistically significant and increased cotton productivity, with improvements ranging from 7% to 74%.

**Fig 15:**
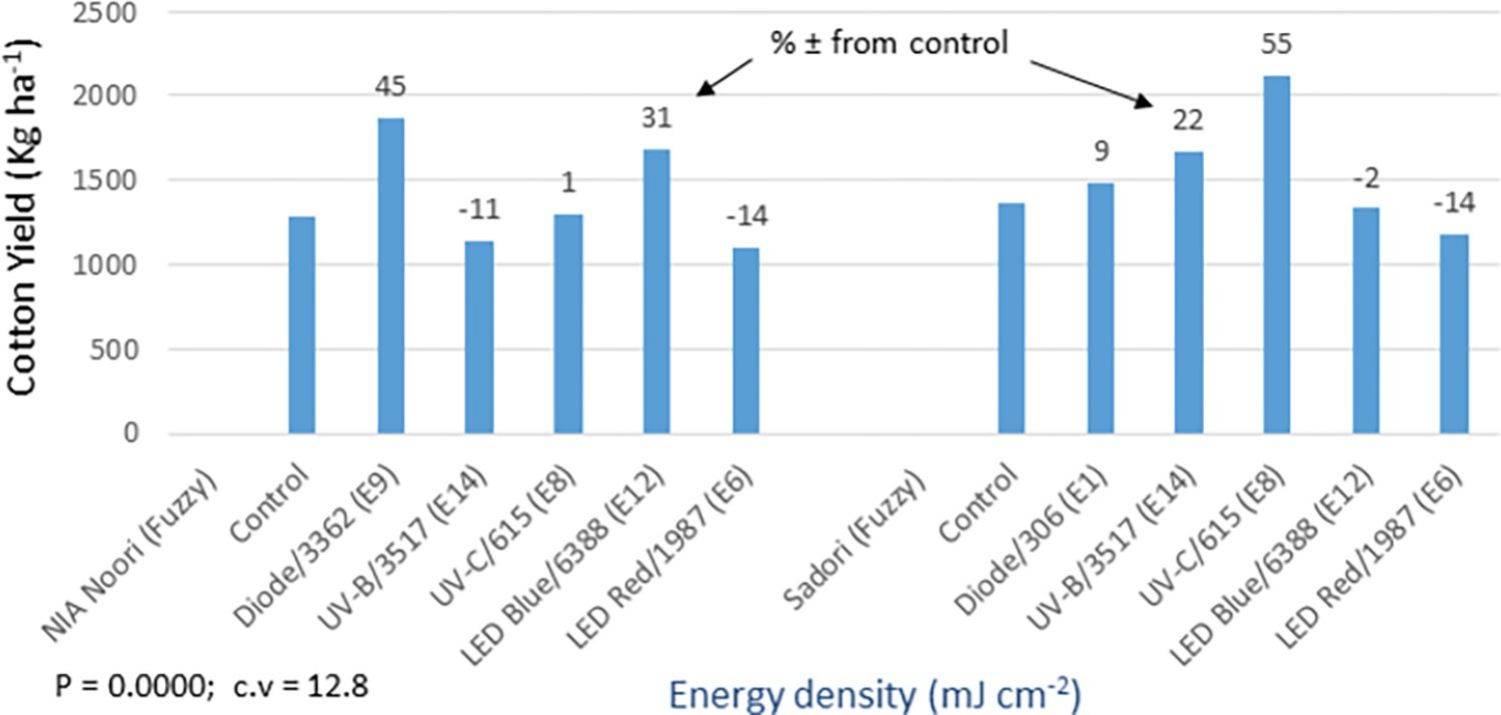
Environment 1 (fuzzy seed trial). Cotton yield (kg ha^-1^) and percent ± in yield over control in field trial conducted at NIA, Tandojam after optical seed priming. LSD, P = 0.0000: Highly significant; cv: Coefficient of variation (%).

The fuzzy seed trial demonstrated a 12–37% increase in germination compared to the control group in NIA-Noori treated seeds, and a 2–11% increase in the case of Sadori variety primed seeds (Fig 14). The statistical analysis of yield data showed significant differences (P = 0.0000) among the light treatments. Specifically, blue LED and diode laser treatments exhibited a 37% and 27% enhanced germination rate over the control, respectively. This, in turn, resulted in a 31% and 45% increase in cotton yield compared to the control (Figs 14 and 15). UV-C and UV-B treatments proved effective for the Sadori variety, showing an 11% and 9% increase in germination over the control, respectively, and similarly improving yield by 55% and 22% compared to the control. Additionally, the laser diode treatment contributed to a 9% increase in cotton productivity over the control. The remaining light treatments did not significantly impact crop productivity for either cotton variety. Based on the percentage increase or decrease (±) in germination and yield compared to the control, the order of effectiveness for different light sources on the four seed types is as follows: blue LED > diode laser > UV-C > UV-B > red LED.

### Environment 2: Faisalabad, Punjab

The city of Faisalabad is located in the Punjab province of Pakistan, at a geographic position of latitude 31°25′00″ N and longitude 73°04′59″ E, at an elevation of 186 meters (610 feet) above sea level [15]. Faisalabad experiences a wet climate, with a total rainfall of 302 mm during the cotton cropping season of 2021 (S4 Table). The rainfall was not evenly distributed throughout the season, with the highest amount occurring in July, reaching 242 mm. The average maximum temperature was observed in June, while the relative humidity was highest in September. The soil texture at the trial site in Faisalabad was classified as loam, consisting of 19.3% sand, 47.3% silt, and 33.4% clay. The soil had an alkaline nature and contained relatively low levels of organic matter (S5 Table). For the fertilizer application, nitrogen was used at a rate of 200 kg ha^-1^, phosphorus at 60 kg ha^-1^, and potash at 100 kg ha^-1^. All the phosphorus and potash, along with one-fourth of the nitrogen, were applied during bed preparation. The remaining nitrogen was split into one-fourth after 30 days, one-fourth at flowering, and one-fourth at the peak boll formation stage (S6 Table). Throughout the season, six irrigations were carried out at different stages of the crop.

**Fig 16:**
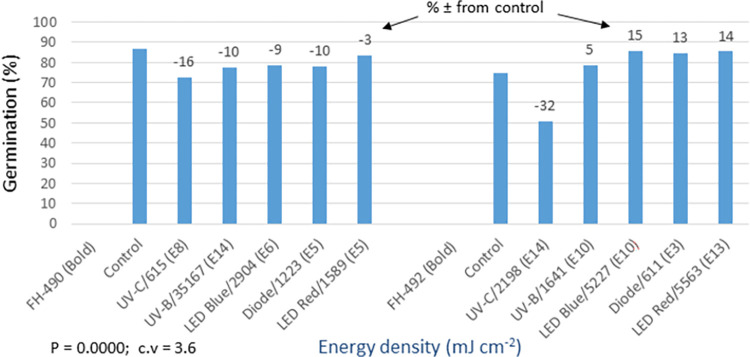
Environment 2 (bold seed trial). Germination (%) and percent ± in germination over control in field trial conducted at CRS, Faisalabad after optical seed priming. LSD, P = 0.0000: Highly significant; cv: Coefficient of variation (%).

**Fig 17:**
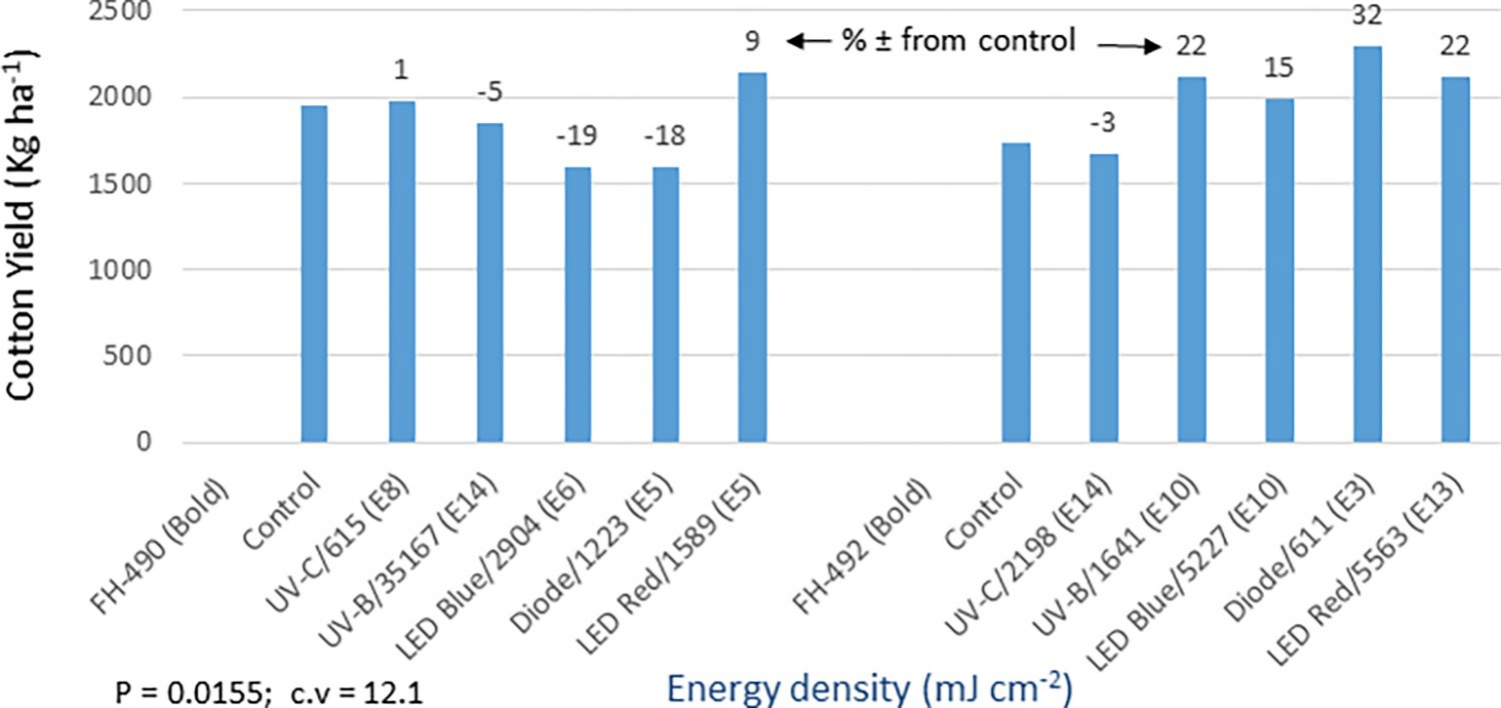
Environment 2 (bold seed trial). Cotton yield (kg ha^-1^) and percent ± in yield over control in field trial conducted at CRS, Faisalabad after optical seed priming. LSD, P = 0.0155: Significant; cv: Coefficient of variation (%).

The analysis of germination (P = 0.000) and yield (P = 0.0155) data showed statistically significant differences among the light treatments in the bold seed trial (Figs 16 and 17). The FH-490 variety did not show any response to the seed priming treatments, except for the red LED treatment, which demonstrated a 9% increase in yield compared to the control. On the other hand, FH-492 exhibited a 5–15% increase in germination and a 15–32% increase in cotton yield compared to the control. All light treatments were effective for this variety, except for UV-C. In addition to the mentioned traits, detailed data on various other morpho-agronomic traits were recorded in both the bold and fuzzy seed trials (S7, S8 Tables). The analysis revealed significant results for all the studied traits in the bold seed trial. It was observed that the improved yield of 9% over the control in FH-490, resulting from the red LED light treatment, was attributed to an increase in the plant population per hectare (PPH) recorded later in the season (S7 Table). Furthermore, the same treatment also led to improvements in cotton quality, such as staple length (SL) and percent ginning outturn (GOT). In the case of FH-492, different seed priming treatments displayed a 15–32% increase in cotton productivity over the control, primarily due to enhancements in morpho-physiological traits, including germination, plant population per hectare (PPH), plant height (PH), number of monopodial branches per plant (MBP), number of sympodial branches per plant (SBP), number of bolls per plant (BP), nodes to first fruiting branch (NFFB), and boll weight (BW). The quality of cotton was also improved in these treatments due to enhancements in quality parameters, such as micronaire (MCN), fiber strength (FS), and percent ginning outturn (GOT).

**Fig 18:**
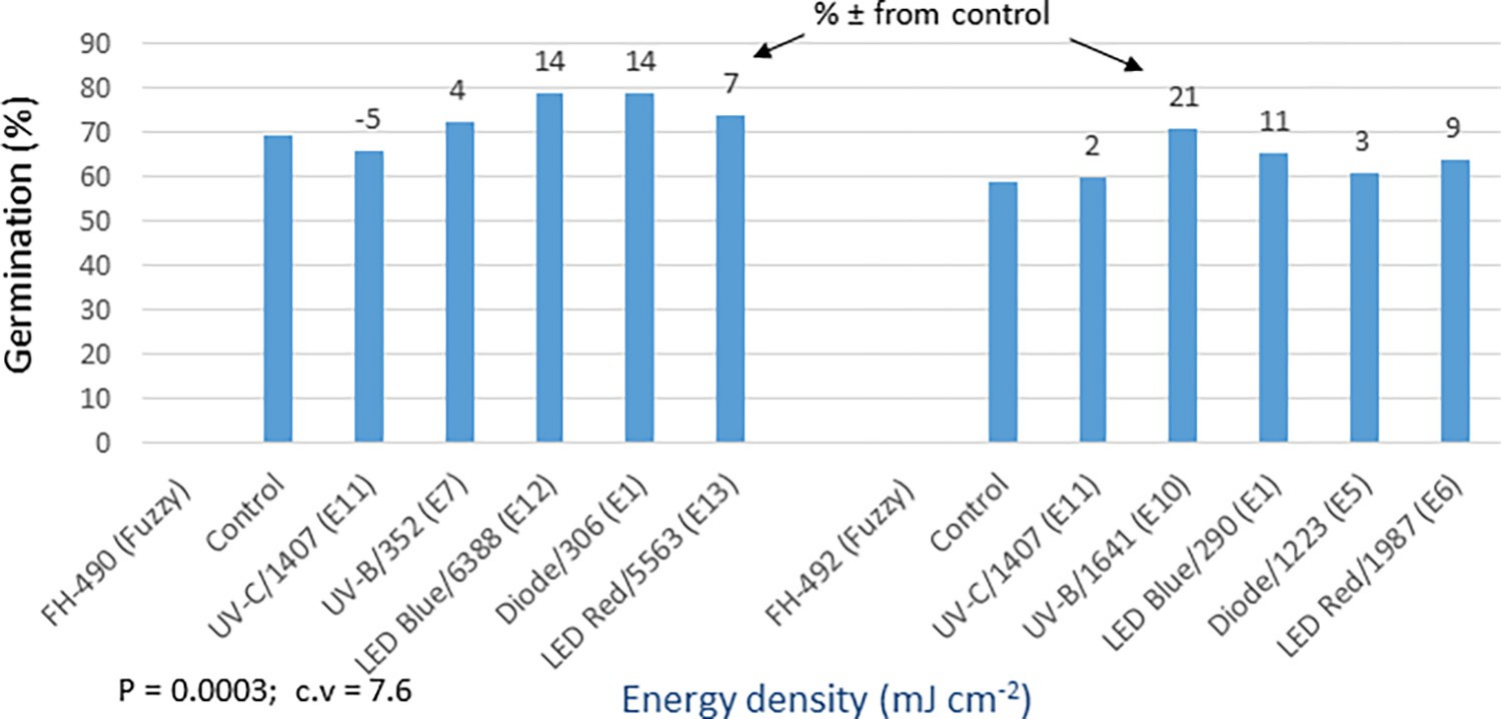
Environment 2 (fuzzy seed trial). (a) Germination (%) and percent ± in germination over control in field trial conducted at CRS, Faisalabad after optical seed priming. LSD, P = 0.0003: Highly significant; cv: Coefficient of variation (%).

**Fig 19:**
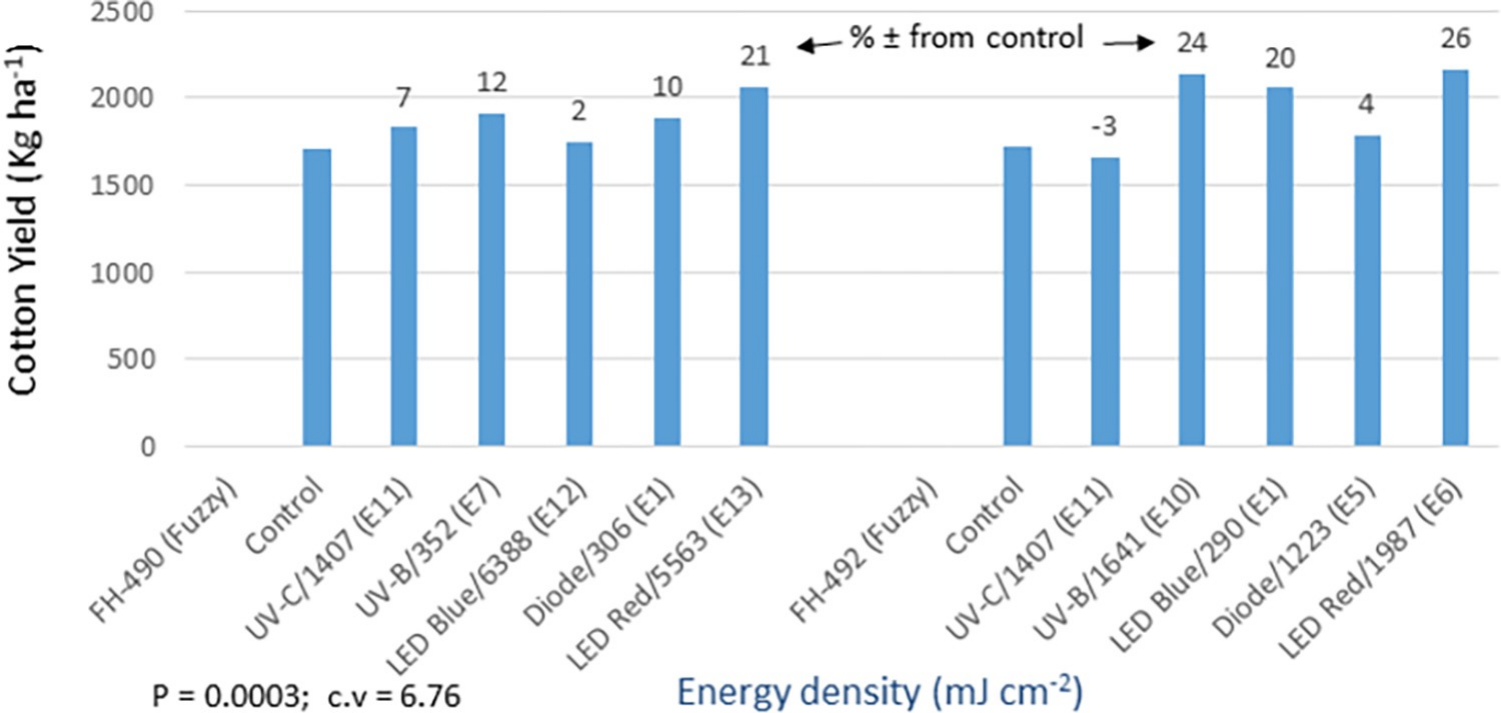
Environment 2 (fuzzy seed trial). Cotton yield (kg ha^-1^) and percent ± in yield over control in field trial conducted at CRS, Faisalabad after optical seed priming. LSD, P = 0.0003: Highly significant; cv: Coefficient of variation (%).

In the fuzzy seed trial, the analysis of germination and yield data displayed highly significant differences (P = 0.0003) among the light treatments (Figs 18 and 19). FH-490 exhibited a 4–14% increase in germination and a 2–21% increase in yield compared to the control. Among the light treatments, the red LED treatment resulted in the highest yield, showing a 21% increase. Similarly, for FH-492, germination showed a 2–21% increase, and yield showed a 4–26% increase over the control. UV-B, blue LED, and red LED treatments performed the best in terms of both assessed traits. The analysis of the detailed data for other morpho-agronomic traits recorded in the fuzzy seed trial mostly yielded significant results (S8 Table). The improvement in cotton yield in response to the optical seed priming treatments was attributed to several traits, including germination, PPH, PH, MBP, SBP, BP, NFFB, and BW. Additionally, the quality of cotton improved due to enhancements in SL, MCN, FS, and GOT traits.

## Discussion

### Laboratory studies

The extensive studies conducted under controlled environments have revealed that the most effective light source for enhancing the germination of cotton seeds is blue LED light, followed by red LED, diode laser, UV-B, and UV-C light. The results have shown that these light sources can lead to a maximum improvement in germination ranging from 67% to 180%. All of these light treatments have displayed promising results in the germination of cotton seeds.

The current study focuses on investigating the effectiveness of pre-sowing optical seed priming techniques in cotton, which is an industrial crop. The literature on the stimulation effects of physical priming agents on cotton seed germination is quite limited. However, a few published papers have reported that treating cotton seeds with combined irradiation of UV-A, UV-B, and UV-C increased germination and growth. This effect was attributed to the activation of two stress-related genes that are involved in dealing with reactive oxygen species (ROS), which are by-products of UV treatment [16]. Therefore, the use of combined UV irradiation was recommended to improve germination and plant growth, especially under stressed conditions. Another technique explored in the study is pulsed electromagnetic treatment of cotton seeds. It was found that this treatment improved germination by 85% compared to the non-irradiated control. Additionally, it enhanced early growth and root parameters when tested under actual field conditions [17].

Yang et al. [18] reported that plants employ photoreceptors to perceive light of different wavelengths and convert it into biological signals, which trigger various responses necessary for plant survival and adaptation in diverse environments. The authors identified three major types of plant photoreceptors: phytochromes (red-light photoreceptors), cryptochromes and phototropins (blue-light photoreceptors), and UVR8 (UV-B photoreceptor). These photoreceptors play a crucial role in regulating plant growth by detecting light wavelengths (colors), intensity, and duration [19]. Among them, the UV-B photoreceptor UVR8 (UV-B resistance locus 8) is responsible for sensing UV-B radiation (280–315 nm). UVR8 is a sensing protein in plants that enhances insect resistance, promotes flavonoid accumulation, and improves photosynthesis efficiency [19]. Moreover, it contributes to plant adaptation and UV-B resistance, which are vital for stress response. UVR8 detects UV-B radiation through light-induced dimer dissociation, which triggers various biological responses involved in light-mediated plant development and protection [18].

The investigation of light-induced seed germination in lettuce has led to the discovery of phytochromes. According to Rye et al. [20], phytochromes exist in two inter-convertible forms, known as Pr and Pfr. When the Pr form absorbs red light, it is converted into the Pfr form. Conversely, when the Pfr form absorbs far-red light, it reverts back to the Pr form. The authors also noted that physiologically, Pfr is the active form of phytochrome, which triggers various plant growth responses, including germination. As a result, exposure to red light stimulates physiological activity, while far-red light inhibits it.

The literature extensively covers the applications of LEDs in food production and preservation. However, their potential use as a seed priming agent is noticeably absent. Previous studies have mainly focused on lasers of various types and UV sources as physical means to improve crop plants. Therefore, this study aimed to investigate the use of LEDs as a pre-sowing seed priming source to improve germination, growth, and yield in cotton crop. The findings indicated that blue LEDs were the most effective source for enhancing cotton crop performance under laboratory conditions.

The present study has revealed promising results regarding the germination of cotton seeds after priming with diode laser treatments. Previous research conducted by various investigators has described the stimulating effects of different lasers on seed germination, growth, and developmental parameters in various crops. For instance, studies on crops like *Isatis indogotica* (Chinese medical plant) [21], soybean [22], Chinese pine seeds [23], sunflower [24], wheat [25–28], white lupine and faba bean [29] and *Moringa oleifera* [30] have demonstrated the positive effects of laser biostimulation on germination rate, seedling growth, physiological traits, and biochemical parameters. These findings align with the results obtained from the present study regarding the enhancement of germination.

Furthermore, seed priming using physical agents such as UV radiation has been reported to have a positive impact on seed germination in economically important crops. Previous studies have shown the beneficial effects of UV radiation treatment on crops like *Vigna mungo* L. [31], kidney bean, cabbage and beet [32], fenugreek [33], wheat [34], *Vigna radiata* [35], black bean [36] and mung bean and groundnut [37]. These investigations have highlighted that the use of UV radiation is an eco-friendly approach to enhance crop germination and growth while providing protection against various stresses. The findings of the current study are consistent with the previous research mentioned above, as they also demonstrate the positive impact of UV irradiation treatments on cotton seed germination.

### Field studies

The analysis of germination and yield data revealed statistically significant differences among the light treatments in the bold and fuzzy seed trials conducted in two contrasting environments: Sindh (NIA Tandojam) and Punjab (CRS Faisalabad) provinces. In Tandojam, the light treatments demonstrated a maximum increase of up to 28% in seed germination and 74% in cotton yield over the control group. Similarly, in Faisalabad, the highest improvements in germination and yield were observed as 15% and 32%, respectively, over the control group. The results of the current studies support earlier research by scientists, who demonstrated that seed priming is an effective method for enhancing germination, stand establishment, crop yield, and tolerance to biotic and abiotic stresses. This method induces various biochemical, physiological, molecular, and subcellular changes in plants [8, 9, 38]. Synchronized germination, resulting from seed priming, leads to increased nutrient uptake and efficient utilization of photosynthetically active radiation (PAR), which in turn enhances the rate of photosynthesis [39]. Ultimately, this leads to improved carboxylation, crop growth, development, and yield [8]. However, the literature on the stimulating effects of pre-sowing treatment of cotton seeds with physical priming agents on plant growth and development is scarce. Nevertheless, some studies have explored this area. One such study found that pre-sowing treatment of cotton seeds with a helium-neon (He-Ne) laser resulted in the highest bio-stimulation, leading to increased plant height and the number of plant blooms [40]. However, it should be noted that He-Ne laser treatment decreased yield and yield components in variety Giza 92, while showing an increase in yield, yield components, and fiber quality traits in variety Ghiza 94 in the M_1_ generation [41]. Another promising approach is the application of Cold Atmospheric-Pressure Plasma (CAP), which is an ionized gas, on cotton seeds before sowing. This treatment has been found to improve seed germination and cold tolerance, with long-lasting effects [42]. It has been suggested that large-scale CAP seed treatment could be a new method to enhance crop establishment in diverse environments.

The effects of pre-sowing laser treatment on seeds of various crops of agricultural importance have been extensively documented in the literature. Previous investigations have shown that seeds of different cultivated crops, when irradiated with a He-Ne (632.8 nm) laser at a power density of 2–5 mW cm^-2^ before sowing, exhibited biostimulation effects leading to improved traits and productivity. Productivity increase was observed in maize (10–20%), spring wheat (20–30%), spring barley (10–25%), sugar beets (10–35%), rape (5–15%), field tomatoes (10–20%) and field cucumbers (10–25%) including improved; plant type, cold tolerance, early maturity, wheat protein (2%) and sugar in sugar beet (15–17%) [43]. Specifically focusing on sugar beet cultivars, seeds were treated with a He-Ne laser (40 mW) using the free falling method. Field experiments revealed an 8–10% increase in root yield, along with higher sugar contents and overall biological yield [44]. Furthermore, pre-sowing treatment of vegetable seeds with a He-Ne laser, performed 3–5 times over a period of 72 to 120 hours, resulted in improved yields and other benefits. Tomatoes showed a yield improvement of 24%, while peppers, cucumbers, onions, and beans exhibited increases of 13%, 15%, 16%, and 27% respectively. Additionally, the treated crops demonstrated early maturity and better product quality [45].

The positive effects of different lasers on various parameters in different crops have been reported by previous researchers. These effects include higher grain yield [46], increased germination percentage [47], improved tolerance to chilling stress [48] in wheat, enhanced germination of seeds [49], increased shoots per square meter, and higher green biomass and dry matter yields in alfalfa [50], higher nutrient contents such as nitrogen (N), calcium (Ca), phosphorus (P), potassium (K), magnesium (Mg), and sodium (Na) in perennial ryegrass [51], increased plant height, number of rows per ear, overall growth, and yield in maize [52], and improved germination, growth, enzymatic function, plant height, number of leaves and panicles, and grain yield in rice [53]. These reports have demonstrated the positive effects of various laser treatments on seed emergence, growth and development, physiological parameters, biochemical properties, and yield productivity in cultivated crops. In this study, pre-sowing seed treatment with a diode laser also resulted in improvements in germination, stand establishment, agro-morphological traits, yield, and quality traits. Therefore, our results corroborate the findings of these previous studies.

The earlier studies have demonstrated that seed treatment with UV radiation is an environmentally friendly method for enhancing crop growth, yield, and stress tolerance. Bilodeau et al. [19] reviewed that blue and UV-A light activate the photoreceptors cryptochrome (320–500 nm) and phototropin, which regulate chloroplast movement, germination, elongation, stomatal opening, enzyme production, crop density, and biotic stress responses. Lower levels of UV radiation affect seed germination, growth architecture, harvest index, chlorophyll synthesis, photosynthesis rate, enzyme functions, ascorbic acid and tocopherol levels, and the development of plant parts [9]. Field-grown tartary buckwheat plants treated with enhanced UV-B radiation exhibited improved plant height and yield [54]. The impact of enhanced UV-B radiation for 111 days on hybrid rice under field conditions resulted in higher leaf chlorophyll and amylose concentrations, photosynthetic activity, tolerance to photoinhibition, decreased diseased grains per panicle, and an improved rate of seed setting [39]. A field experiment showed that UV-C seed treatment for up to 60 minutes increased germination percentage, seedling growth parameters including seedling vigor, and groundnut productivity (biomass and yield) compared to the control [55]. Treating cabbage seeds with a low UV-C dose of 3.6 kJ m^-2^ reduced the population density of fungus in infected leaves, improved weight and head diameter, and resulted in a 60% reduction in disease severity when stored for 8 months at room temperature [56]. These studies have revealed that UV irradiation in various crops has beneficial effects on physiological, biochemical, and morphological parameters, contributing to increased growth and yield of agricultural crops. The present study also corroborated earlier findings by demonstrating improvements in crop germination, stand and growth, cotton yield and yield-related traits, and fiber quality in response to UV-B and UV-C seed treatments.

The detailed study of the two trials conducted at Faisalabad revealed that PPH (plant population per hectare) is a major contributing factor to improved yields. Therefore, it has been inferred that in addition to recording germination at the early stages of crop development, PPH must also be recorded later in the crop season. This parameter will cover the plants that germinate late and provide a more accurate picture of crop establishment. In agriculture, priming approaches hold special importance as they enhance crop seedling establishment under adverse environmental conditions [17]. These approaches help improve the overall performance of crops in challenging circumstances. When considering the percentage change in germination and yield over the control group in the bold and fuzzy seed trials at Faisalabad, it was observed that different light sources had varying contributions. The order of contribution was found to be red LED > UV-B > blue LED > diode laser > UV-C.

The weather conditions in both tested environments exhibited variations in terms of rainfall, temperature, and humidity. Tandojam was characterized as a dry environment, while Faisalabad was comparatively wet, with higher temperatures and relative humidity during the 2021 cropping season. When comparing the trials, it was observed that germination rates were higher in Faisalabad, primarily due to the planting of three seeds per hill, in contrast to Tandojam where only a single seed was sown per hill. Additionally, the results varied due to the different cotton varieties planted in both environments. This inconsistency arises from the fact that only the approved varieties designated for general cultivation in those specific locations are permitted to be planted there. Varietal response was noted in relation to the effects of irradiation from various light sources on seed germination and yield. The findings from the trials conducted in both environments revealed that the most effective light sources for optical seed priming were blue LED and diode laser, followed by UV-B, red LED, and UV-C, for enhancing germination and yield in cotton crop. The hypothesis of the current study has been proven, indicating that seed priming using different colors of light improves cotton seed germination, stand establishment, growth, productivity, and quality in diverse environments. The aforementioned field results are encouraging, showcasing significant improvements in germination, yield, and related traits, as well as the quality of cotton crops. This highlights the potential of seed priming technology and emphasizes the need for further research in this area.

The use of reported optical seed priming is particularly valuable for commercial and especially for organic farming, where chemicals, hormones, and extracts are not allowed. Moreover, the application of these priming agents is difficult and impractical for agricultural use, while optical seed priming technology is simple, rapid, and cost-effective. Farmers will readily adopt the proposed method as soon as it is commercialized. Investigations have also begun on the okra vegetable (lady’s finger), showing promising results with the use of blue LED light. Further research is underway using other light sources. A low-cost device has been proposed, consisting of a top light source and a base made of a conveyor belt for seed irradiation. The device will have adjustable speed and exposure timings. Once the technique is refined, there are plans to distribute the low-cost optical technology and portable commercial device among farming communities and commercial seed companies.

## Conclusion

The investigation into the impact of optical seed priming technology on the germination of cotton under laboratory conditions revealed significant improvements. Various light sources were tested, and the maximum enhancement in germination ranged from 67–180%. The most effective light source was determined to be blue LED light, followed by red LED, diode laser, UV-B, and UV-C light. All of these light treatments showed promise in promoting cotton seed germination. Evaluating the effects of optical seed priming technology on cotton performance at two different agro-ecological sites showed significant improvements in germination and yield compared to the control group. At the Tandojam site, germination and yield increased by up to 37% and 74% respectively, while at the Faisalabad site, the increases were up to 21% and 32% respectively. The variation in field results between the two environments can be attributed to the use of different cotton varieties approved for cultivation in those specific locations. In the Faisalabad environment, the improvement in cotton yield resulting from optical seed priming treatments was associated with various traits such as germination, plant height (PH), no. of monopodial branches per plant (MBP), no. of sympodial branches per plant (SBP), no. of bolls per plant (BP), nodes to first fruiting branch (NFFB), and boll weight (BW). Additionally, the quality of cotton improved with enhancements observed in staple length (SL), micronaire value (MCN), fiber strength (FS), and ginning outturn (GOT) traits. Based on the trial results, the most effective light sources for promoting germination and yield in cotton were found to be blue LED and diode laser, followed by UV-B, red LED, and UV-C. The hypothesis of the study, which proposed that seed priming using light of different colors improves cotton seed germination, stand establishment, plant growth, productivity, and quality in varying environments, was supported by the findings. The field results indicate the potential of seed priming technology for commercial application in cotton cultivation.

## Supporting information

S1 Table Weather data of cotton growing season at environment 1, Tandojam (Sindh) for 2021. Source: Pakistan Meteorological Department, Tandojam, Pakistan.
(DOCX)

S2 Table Analytical results of soil samples from cotton trials at Tandojam during 2021. Soil Texture = Clay Loam (Sand = 22.50%, Silt = 41.34% and Clay = 36.16%). Source: Soil & Environmental Sciences Division, NIA Tando Jam.
(DOCX)

S3 Table Agronomic practices for cotton trials at Tandojam during 2021. Weedicide spray: Dual Gold = 07.05.2021, Insecticide spray: Polytrin C = 05.07.2021 and 28.07.2021, Manual weed control = 01.06.2021, Tractor ploughing (for weed removal) = 18.06.2021, 1st Cotton picking = 31.08.2021, 2nd Cotton picking = 30.09.2021.
(DOCX)

S4 Table Weather data of cotton growing season at environment 2, Faisalabad (Punjab) for 2021. Note: Mean Mini. Temp: Mean Minimum Temperature; Mean Max. Temp: Mean Maximum Temperature; R.H. = Relative Humidity. Source: Plant physiology section, Agronomic Research Institute, AARI, Faisalabad.
(DOCX)

S5 Table Analytical results of soil samples from cotton trials at Faisalabad during 2021. Soil Texture = Loam (Sand = 19.30%, Silt = 47.3%, and Clay = 33.40%). Source: Soil & Water Testing, AARI, Faisalabad.
(DOCX)

S6 Table Agronomic practices for cotton trials at Faisalabad during 2021. Fertilizer Application @ Nitrogen = 200 kg ha^-1^, Phosphorus = 60 kg ha^-1^, Potash = 100 kg ha^-1^. All the phosphorus and potash, and 1/4th of nitrogen was applied at bed preparation. Remaining nitrogen splits into 1/4th after 30 days, 1/4th at flowering and 1/4th at peak boll formation.
(DOCX)

S7 Table Environment 2 (bold seed trial). Detailed data of morpho-agronomic traits of the field trial conducted at CRS, Faisalabad after optical seed priming. DFB = Days to first bud, DFF = Days to first flower, PPH = Plant population per hectare, SL = Staple length (mm), MCN = Micronaire (μg/in), FS = Fiber strength (g/tex), GOT = Ginning out turn (%), PH = Plant height (cm), MBP = No. of monopodial branches per plant, SBP = No. of sympodial branches per plant, BP = No. of bolls per plant, NFFB = Nodes to first fruiting branch, BW = Boll weight (gm).
(DOCX)

S8 Table Environment 2 (fuzzy seed trial). Detailed data of morpho-agronomic traits of the field trial conducted at CRS, Faisalabad after optical seed priming. DFB = Days to first bud, DFF = Days to first flower, PPH = Plant population per hectare, SL = Staple length (mm), MCN = Micronaire (μg/in), FS = Fiber strength (g/tex), GOT = Ginning out turn (%), PH = Plant height (cm), MBP = No. of monopodial branches per plant, SBP = No. of sympodial branches per plant, BP = No. of bolls per plant, NFFB = Nodes to first fruiting branch, BW = Boll weight (gm).
(DOCX)

S9 Table Germination (%) and percent increase in germination over non-irradiated control after irradiation of seeds with LED blue light in controlled environment. (DOCX)

S10 Table Germination (%) and percent increase in germination over control after seed irradiation with LED red light in controlled environment. (DOCX)

S11 Table Germination (%) and percent increase in germination over control after seed irradiation with diode laser in controlled environment. (DOCX)

S12 Table Germination (%) and percent increase in germination over control after seed irradiation with UV-B light in controlled environment. (DOCX)

S13 Table Germination (%) and percent increase in germination over control after seed irradiation with UV-C light in controlled environment. (DOCX)

S14 Table Environment 1 (bold seed trial). Germination (%) and percent increase/decrease (±) in germination over control, and Cotton yield (kg ha^-1^) and percent ± in yield over control in field trial conducted at NIA, Tandojam after optical seed priming.
(DOCX)

S15 Table Environment 1 (fuzzy seed trial). Germination (%) and percent ± in germination over control and Cotton yield (kg ha^-1^) and percent ± in yield over control in field trial conducted at NIA, Tandojam after optical seed priming.
(DOCX)

## Abbreviations

LED: Light Emitting Diode
DFB: days to first bud
DFF: days to first flower
PPH: plant population per hectare
SL: staple length
MCN: micronaire
FS: fiber strength
GOT: ginning out turn
PH: plant height
MBP: no. of monopodial branches per plant
SBP: no. of sympodial branches per plant
BP: no. of bolls per plant
NFFB: nodes to first fruiting branch
BW: boll weight
UV: ultraviolet
ROS: reactive oxygen species
