# Supplementary material for: Enhancement of germination and yield of cotton through optical seed priming: Lab. and diverse environment studies
Source: PLoS One. 2023 Jul 20;18(7):e0288255. doi: 10.1371/journal.pone.0288255 (PMC10358893; doi:10.1371/journal.pone.0288255)
Supplement: S1 Table — Source: Pakistan Meteorological Department, Tandojam, Pakistan. (DOCX) [file pone.0288255.s001.docx]

**Supporting Information**

**S1 Table. Weather data of cotton growing season at environment 1, Tandojam (Sindh) for 2021.**

| Month | Total Rainfall  (mm) | Air Temp. (^o^C)  Min. | Air Temp. (^o^C)  Max. | Air Temp (^o^C)  Mean | Soil Temp. (^o^C)  Mean | Relative Humidity (%)  Mean | Sunshine (Hrs) Mean |
| --- | --- | --- | --- | --- | --- | --- | --- |
| May | 4.0 | 23.9 | 40.0 | 31.9 | 36.9 | 50 | 9.26 |
| June | 0.0 | 26.0 | 39.1 | 32.6 | 38.4 | 57 | 9.26 |
| July | 19.0 | 26.8 | 36.8 | 31.7 | 37.9 | 64 | 6.79 |
| August | 4.0 | 25.5 | 35.7 | 30.5 | 35.4 | 63 | 8.65 |
| September | 12.5 | 26.2 | 37.5 | 31.8 | 36.2 | 63 | 7.25 |
| October | 0.0 | 20.6 | 35.8 | 28.2 | 30.7 | 53 | 8.27 |
| November | 0.0 | 14.3 | 30.0 | 22.1 | 22.0 | 53 | 7.45 |

Source: Pakistan Meteorological Department, Tandojam, Pakistan
