# Supplementary material for: Enhancement of germination and yield of cotton through optical seed priming: Lab. and diverse environment studies
Source: PLoS One. 2023 Jul 20;18(7):e0288255. doi: 10.1371/journal.pone.0288255 (PMC10358893; doi:10.1371/journal.pone.0288255)
Supplement: S2 Table — Soil Texture = Clay Loam (Sand = 22.50%, Silt = 41.34% and Clay = 36.16%). Source: Soil & Environmental Sciences Division, NIA Tando Jam. (DOCX) [file pone.0288255.s002.docx]

**S2 Table. Analytical results of soil samples from cotton trials at Tandojam during 2021.**

| Sr. No. | Soil parameter | Unit | Value |
| --- | --- | --- | --- |
| 1 | pH | - | 7.90 |
| 2 | EC | dSm^-1^ | 2.3 |
| 3 | Total N | % | 0.058 |
| 4 | Available P | mg kg^-1^ | 3.67 |
| 5 | Available K | mg kg^-1^ | 170 |
| 6 | Organic Matter | % | 0.69 |

Soil Texture = Clay Loam (Sand = 22.50%, Silt = 41.34% and Clay = 36.16%)

Source: Soil & Environmental Sciences Division, NIA Tando Jam
