# Supplementary material for: Enhancement of germination and yield of cotton through optical seed priming: Lab. and diverse environment studies
Source: PLoS One. 2023 Jul 20;18(7):e0288255. doi: 10.1371/journal.pone.0288255 (PMC10358893; doi:10.1371/journal.pone.0288255)
Supplement: S3 Table — Weedicide spray: Dual Gold = 07.05.2021, Insecticide spray: Polytrin C = 05.07.2021 and 28.07.2021, Manual weed control = 01.06.2021, Tractor ploughing (for weed removal) = 18.06.2021, 1st Cotton picking = 31.08.2021, 2nd Cotton picking = 30.09.2021. (DOCX) [file pone.0288255.s003.docx]

**S3 Table. Agronomic practices for cotton trials at Tandojam during 2021.**

| Date | Fertilizer @ | Date | Irrigation |
| --- | --- | --- | --- |
| 06.05.2021 | DAP 0.5 bag | 12.05.2021 | 1^st^ |
| 24.05.2021 | Urea 0.5 bag | 24.05.2021 | 2^nd^ |
|  |  | 07.06.2021 | 3^rd^ |
| 12.06.2021 | Urea 0.5 bag | 13.06.2021 | 4^th^ |
|  |  | 26.06.2021 | 5^th^ |
| 08.07.2021 | Urea 0.5 bag | 08.07.2021 | 6^th^ |
|  |  | 30.07.2021 | 7^th^ |
|  |  | 11.08.2021 | 8^th^ |

Weedicide spray: Dual Gold = 07.05.2021

Insecticide spray : Polytrin C = 05.07.2021 and 28.07.2021

Manual weed control = 01.06.2021

Tractor ploughing (for weed removal) = 18.06.2021

1^st^ Cotton picking = 31.08.2021

2^nd^ Cotton picking = 30.09.2021
