# Supplementary material for: Enhancement of germination and yield of cotton through optical seed priming: Lab. and diverse environment studies
Source: PLoS One. 2023 Jul 20;18(7):e0288255. doi: 10.1371/journal.pone.0288255 (PMC10358893; doi:10.1371/journal.pone.0288255)
Supplement: S4 Table — Note: Mean Mini. Temp: Mean Minimum Temperature; Mean Max. Temp: Mean Maximum Temperature; R.H. = Relative Humidity. Source: Plant physiology section, Agronomic Research Institute, AARI, Faisalabad. (DOCX) [file pone.0288255.s004.docx]

**S4 Table. Weather data of cotton growing season at environment 2, Faisalabad (Punjab) for 2021.**

| Months | Rainfall (mm) | Mean mini. Temp. (^º^C) | Mean max. Temp. (^º^C) | Mini. Temp. (^º^C) | Max. Temp. (^º^C) | Mean mini.  R. H. (%) | Mean max.  R. H. (%) | Mini.  R. H. (%) | Max.  R. H. (%) |
| --- | --- | --- | --- | --- | --- | --- | --- | --- | --- |
| May | 11.2 | 24.0 | 38.6 | 27.0 | 43.5 | 36.1 | 54.4 | 25 | 91 |
| June | 14.2 | 25.6 | 39.0 | 20.5 | 42.5 | 40.6 | 60.2 | 27 | 72 |
| July | 241.6 | 26.9 | 37.3 | 22.0 | 42.5 | 57.0 | 69.0 | 30 | 92 |
| August | 5.0 | 26.8 | 37.7 | 24.0 | 40.5 | 52.5 | 69.4 | 36 | 85 |
| September | 29.8 | 25.5 | 35.2 | 22.5 | 38.5 | 58.3 | 73.7 | 45 | 88 |
| October | 0.3 | 19.0 | 33.7 | 13.0 | 38.0 | 47.4 | 70.0 | 32 | 85 |
| November | 0.0 | 11.0 | 28.3 | 7.0 | 32.0 | 47.5 | 81.3 | 39 | 94 |

Note: Mean Mini. Temp: Mean Minimum Temperature; Mean Max. Temp: Mean Maximum Temperature; R.H. = Relative Humidity

Source: Plant physiology section, Agronomic Research Institute, AARI, Faisalabad.
