# Supplementary material for: Enhancement of germination and yield of cotton through optical seed priming: Lab. and diverse environment studies
Source: PLoS One. 2023 Jul 20;18(7):e0288255. doi: 10.1371/journal.pone.0288255 (PMC10358893; doi:10.1371/journal.pone.0288255)
Supplement: S5 Table — Soil Texture = Loam (Sand = 19.30%, Silt = 47.3%, and Clay = 33.40%). Source: Soil & Water Testing, AARI, Faisalabad. (DOCX) [file pone.0288255.s005.docx]

**S5 Table. Analytical results of soil samples from cotton trials at Faisalabad during 2021.**

| Sr. No. | Soil parameter | Unit | Value |
| --- | --- | --- | --- |
| 1 | pH | - | 8.2 |
| 2 | EC | dSm^-1^ | 2.4 |
| 3 | Total N | % | 0.070 |
| 4 | Available P | mg kg^-1^ | 4.3 |
| 5 | Available K | mg kg^-1^ | 150 |
| 6 | Organic Matter | % | 0.36 |

Soil Texture = Loam (Sand = 19.30%, Silt = 47.3%, and Clay = 33.40%)

Source: Soil & Water Testing, AARI, Faisalabad
