# Supplementary material for: Enhancement of germination and yield of cotton through optical seed priming: Lab. and diverse environment studies
Source: PLoS One. 2023 Jul 20;18(7):e0288255. doi: 10.1371/journal.pone.0288255 (PMC10358893; doi:10.1371/journal.pone.0288255)
Supplement: S6 Table — Fertilizer Application @ Nitrogen = 200 kg ha-1, Phosphorus = 60 kg ha-1, Potash = 100 kg ha-1. All the phosphorus and potash, and 1/4th of nitrogen was applied at bed preparation. Remaining nitrogen splits into 1/4th after 30 days, 1/4th at flowering and 1/4th at peak boll formation. (DOCX) [file pone.0288255.s006.docx]

**S6 Table. Agronomic practices for cotton trials at Faisalabad during 2021.**

**Fertilizer Application @**

Nitrogen = 200 kg ha^-1^

Phosphorus = 60 kg ha^-1^

Potash = 100 kg ha^-1^

All the phosphorus and potash, and 1/4th of nitrogen was applied at bed preparation.

Remaining nitrogen splits into 1/4th after 30 days, 1/4th at flowering and 1/4th at peak boll formation.

**Irrigation Application**

| Flood irrigation stage | No. |
| --- | --- |
| At the time of sowing | 1^st^ |
| After emergence and gap filling | 2^nd^ |
| At square initiation | 3^rd^ |
| At start of flowering | 4^th^ |
| At peak flowering stage | 5^th^ |
| At boll maturation stage | 6^th^ |
