# Supplementary material for: Enhancement of germination and yield of cotton through optical seed priming: Lab. and diverse environment studies
Source: PLoS One. 2023 Jul 20;18(7):e0288255. doi: 10.1371/journal.pone.0288255 (PMC10358893; doi:10.1371/journal.pone.0288255)
Supplement: S7 Table — Detailed data of morpho-agronomic traits of the field trial conducted at CRS, Faisalabad after optical seed priming. DFB = Days to first bud, DFF = Days to first flower, PPH = Plant population per hectare, SL = Staple length (mm), MCN = Micronaire (μg/in), FS = Fiber strength (g/tex), GOT = Ginning out turn (%), PH = Plant height (cm), MBP = No. of monopodial branches per plant, SBP = No. of sympodial branches per plant, BP = No. of bolls per plant, NFFB = Nodes to first fruiting branch, BW = Boll weight (gm). (DOCX) [file pone.0288255.s007.docx]

**S7 Table. Environment 2 (Bold seed trial). Detailed data of morpho-agronomic traits of the field trial conducted at CRS, Faisalabad after optical seed priming.**

| Variety/  Seed type | Treatment | Energy density  (mJ cm^-2^) | **Germi-nation** | **% ± from control** | DFB | DFF | PPH | SL | MCN | FS | GOT | PH | MBP | SBP | BP | NFFB | BW | Yield Kg ha^-1^ | **% ± from control** |
| --- | --- | --- | --- | --- | --- | --- | --- | --- | --- | --- | --- | --- | --- | --- | --- | --- | --- | --- | --- |
| FH-490,  Bold seed | 1. Control | Control | 87 | 0 | 34 | 51 | 31322 | 24.82 | 5.01 | 30.38 | 39.5 | 105 | 3 | 17 | 30 | 7 | 3.20 | 1954 | 0 |
|  | 2. UV-C | 615 (E8) | 73 | -16 | 35 | 53 | 30844 | 25.29 | 4.71 | 26.61 | 40.3 | 108 | 4 | 19 | 30 | 5 | 3.08 | 1975 | 1 |
|  | 3. UV-B | 35167 (E14) | 78 | -10 | 35 | 54 | 31082 | 24.00 | 4.76 | 30.15 | 40.0 | 102 | 2 | 15 | 26 | 7 | 3.18 | 1851 | -5 |
|  | 4. LED Blue | 2904 (E6) | 79 | -9 | 33 | 53 | 29648 | 27.08 | 4.67 | 31.29 | 42.2 | 91 | 2 | 16 | 17 | 6 | 2.99 | 1591 | -19 |
|  | 5. Diode Laser | 1223 (E5) | 78 | -10 | 35 | 53 | 29170 | 26.04 | 4.65 | 31.96 | 40.3 | 101 | 2 | 20 | 28 | 7 | 3.09 | 1595 | -18 |
|  | 6. LED Red | 1589 (E5) | 84 | -3 | 34 | 51 | 35864 | 25.20 | 4.88 | 28.36 | 40.2 | 84 | 1 | 16 | 13 | 5 | 3.06 | 2139 | 9 |
| FH-492,  Bold seed | 7. Control | Control | 75 | 0 | 35 | 52 | 29887 | 25.47 | 4.99 | 26.93 | 40.3 | 73 | 2 | 15 | 15 | 7 | 2.95 | 1731 | 0 |
|  | 8. UV-C | 2198 (E14) | 51 | -32 | 36 | 53 | 26540 | 25.95 | 4.99 | 26.76 | 38.9 | 92 | 1 | 17 | 22 | 8 | 3.09 | 1671 | -3 |
|  | 9. UV-B | 1641 (E10) | 78 | 5 | 35 | 53 | 33952 | 25.18 | 5.10 | 29.56 | 41.8 | 98 | 2 | 20 | 27 | 5 | 3.06 | 2112 | 22 |
|  | 10. LED Blue | 5227 (E10) | 86 | 15 | 35 | 53 | 33473 | 25.05 | 5.04 | 27.69 | 41.1 | 94 | 1 | 18 | 25 | 9 | 3.02 | 1988 | 15 |
|  | 11. Diode Laser | 611 (E3) | 84 | 13 | 36 | 54 | 35864 | 24.49 | 5.21 | 28.00 | 40.5 | 104 | 1 | 21 | 29 | 8 | 3.03 | 2291 | 32 |
|  | 12. LED Red | 5563 (E13) | 85 | 14 | 36 | 53 | 35625 | 24.55 | 5.19 | 24.95 | 41.4 | 105 | 2 | 21 | 24 | 7 | 2.93 | 2112 | 22 |
|  | ANOVA | P value: | 0.0000 | - | 0.0635 | 0.0347 | 0.0002 | 0.0000 | 0.0018 | 0.0000 | 0.0148 | 0.0000 | 0.0001 | 0.0000 | 0.0000 | 0.0006 | 0.0028 | 0.0155 | - |
|  | Coefficient of variation (cv): | | 3.6 | - | 3.1 | 2.2 | 6.8 | 1.5 | 3.3 | 2.1 | 2.3 | 4.9 | 36.7 | 6.1 | 6.5 | 14.2 | 2.3 | 12.1 | - |

DFB = Days to first bud, DFF = Days to first flower, PPH = Plant population per hectare, SL = Staple length (mm), MCN = Micronaire (µg/in), FS = Fiber strength (g/tex), GOT = Ginning out turn (%), PH = Plant height (cm), MBP = No. of monopodial branches per plant, SBP = No. of sympodial branches per plant, BP = No. of bolls per plant, NFFB = Nodes to first fruiting branch, BW = Boll weight (gm).
