# Supplementary material for: Enhancement of germination and yield of cotton through optical seed priming: Lab. and diverse environment studies
Source: PLoS One. 2023 Jul 20;18(7):e0288255. doi: 10.1371/journal.pone.0288255 (PMC10358893; doi:10.1371/journal.pone.0288255)
Supplement: S8 Table — Detailed data of morpho-agronomic traits of the field trial conducted at CRS, Faisalabad after optical seed priming. DFB = Days to first bud, DFF = Days to first flower, PPH = Plant population per hectare, SL = Staple length (mm), MCN = Micronaire (μg/in), FS = Fiber strength (g/tex), GOT = Ginning out turn (%), PH = Plant height (cm), MBP = No. of monopodial branches per plant, SBP = No. of sympodial branches per plant, BP = No. of bolls per plant, NFFB = Nodes to first fruiting branch, BW = Boll weight (gm). (DOCX) [file pone.0288255.s008.docx]

**S8 Table. Environment 2 (Fuzzy seed trial). Detailed data of morpho-agronomic traits of the field trial conducted at CRS, Faisalabad after optical seed priming.**

| Variety/  Seed type | Treatment | Energy density (mJ cm^-2^) | **Germi-nation** | **% ± from control** | DFB | DFF | PPH | SL | MCN | FS | GOT | PH | MBP | SBP | BP | NFFB | BW | Yield Kg ha^-1^ | **% ± from control** |
| --- | --- | --- | --- | --- | --- | --- | --- | --- | --- | --- | --- | --- | --- | --- | --- | --- | --- | --- | --- |
| FH-490, Fuzzy seed | 1. Control | Control | 69 | 0 | 36 | 52 | 27974 | 24.87 | 4.32 | 30.42 | 38.8 | 88 | 3 | 15 | 24 | 6 | 3.11 | 1712 | 0 |
|  | 2. UV-C | 1407 (E11) | 66 | -5 | 35 | 53 | 28931 | 24.84 | 4.69 | 28.35 | 40.1 | 86 | 3 | 16 | 28 | 7 | 3.13 | 1831 | 7 |
|  | 3. UV-B | 352 (E7) | 72 | 4 | 35 | 53 | 29170 | 24.28 | 4.68 | 27.22 | 40.3 | 99 | 1 | 17 | 20 | 9 | 3.21 | 1915 | 12 |
|  | 4. LED Blue | 6388 (E12) | 79 | 14 | 36 | 53 | 28692 | 25.65 | 5.04 | 31.69 | 39.7 | 95 | 3 | 16 | 25 | 8 | 3.12 | 1743 | 2 |
|  | 5. Diode Laser | 306 (E1) | 79 | 14 | 35 | 52 | 29887 | 26.71 | 4.70 | 30.93 | 39.7 | 98 | 1 | 16 | 20 | 7 | 3.14 | 1891 | 10 |
|  | 6. LED Red | 5563 (E13) | 74 | 7 | 35 | 53 | 30604 | 24.12 | 4.95 | 27.60 | 40.6 | 118 | 2 | 22 | 30 | 9 | 2.99 | 2064 | 21 |
| FH-492, Fuzzy seed | 7. Control | Control | 59 | 0 | 35 | 52 | 22714 | 23.92 | 5.35 | 25.89 | 40.7 | 102 | 1 | 22 | 27 | 7 | 2.97 | 1719 | 0 |
|  | 8. UV-C | 1407 (E11) | 60 | 2 | 36 | 54 | 22236 | 26.44 | 5.14 | 27.92 | 39.0 | 101 | 1 | 18 | 29 | 7 | 3.15 | 1663 | -3 |
|  | 9. UV-B | 1641 (E10) | 71 | 21 | 35 | 53 | 28931 | 25.37 | 5.06 | 27.32 | 41.4 | 97 | 2 | 18 | 28 | 9 | 2.90 | 2135 | 24 |
|  | 10. LED Blue | 290 (E1) | 65 | 11 | 36 | 54 | 28452 | 25.00 | 5.43 | 29.49 | 39.6 | 103 | 0 | 21 | 25 | 9 | 3.22 | 2056 | 20 |
|  | 11. Diode Laser | 1223 (E5) | 61 | 3 | 35 | 53 | 23670 | 25.14 | 4.88 | 29.33 | 42.4 | 104 | 1 | 21 | 31 | 8 | 2.97 | 1788 | 4 |
|  | 12. LED Red | 1987 (E6) | 64 | 9 | 36 | 54 | 27018 | 25.85 | 5.02 | 27.29 | 40.8 | 100 | 2 | 22 | 28 | 9 | 3.03 | 2164 | 26 |
|  | ANOVA | P value: | 0.0003 | - | 0.9892 | 0.5400 | 0.0000 | 0.0000 | 0.0000 | 0.0000 | 0.0000 | 0.0000 | 0.0001 | 0.0001 | 0.0000 | 0.0073 | 0.0468 | 0.0003 | - |
|  | Coefficient of variation (cv): | | 7.6 | - | 3.8 | 2.5 | 5.4 | 1.1 | 3.1 | 2.7 | 1.5 | 3.4 | 40.8 | 9.4 | 4.5 | 14.8 | 3.9 | 6.8 | - |

DFB = Days to first bud, DFF = Days to first flower, PPH = Plant population per hectare, SL = Staple length (mm), MCN = Micronaire (µg/in), FS = Fiber strength (g/tex), GOT = Ginning out turn (%), PH = Plant height (cm), MBP = No. of monopodial branches per plant, SBP = No. of sympodial branches per plant, BP = No. of bolls per plant, NFFB = Nodes to first fruiting branch, BW = Boll weight (gm).
