# Supplementary material for: Enhancement of germination and yield of cotton through optical seed priming: Lab. and diverse environment studies
Source: PLoS One. 2023 Jul 20;18(7):e0288255. doi: 10.1371/journal.pone.0288255 (PMC10358893; doi:10.1371/journal.pone.0288255)
Supplement: S9 Table — (DOCX) [file pone.0288255.s009.docx]

**S9 Table (a, b). Germination (%) and percent increase in germination over non-irradiated control after irradiation of seeds with LED blue light in controlled environment.**

**(a)**

| **Variety/**  **Seed type** | **Exposure** | **Exposure time (minutes)** | **Energy density (mJ cm^-2^)** | **Mean**  **Germination** | **% ± from control** | **S.E** |
| --- | --- | --- | --- | --- | --- | --- |
| Cyto-124, Bold seed | Control | Control | Control | 25 | - | 3.5 |
|  | E1 | 1.0 | 290 | 40 | 60 | 2.0 |
|  | E2 | 2.0 | 581 | 35 | 40 | 3.5 |
|  | E5 | 8.0 | 2323 | 40 | 60 | 4.1 |
|  | E6 | 10.0 | 2904 | 45 | 80 | 3.5 |
|  | E8 | 14.0 | 4065 | 35 | 40 | 3.5 |
|  | E10 | 18.0 | 5227 | 35 | 40 | 3.5 |
|  | E11 | 20.0 | 5807 | 35 | 40 | 3.5 |
|  | E12 | 22.0 | 6388 | 45 | 80 | 3.5 |
| SADORI, Fuzzy seed | Control | Control | Control | 30 | - | 5.0 |
|  | E1 | 1.0 | 290 | 43 | 42 | 2.5 |
|  | E2 | 2.0 | 581 | 38 | 25 | 2.5 |
|  | E5 | 8.0 | 2323 | 38 | 25 | 5.0 |
|  | E6 | 10.0 | 2904 | 38 | 25 | 2.5 |
|  | E10 | 18.0 | 5227 | 38 | 25 | 2.5 |
|  | E12 | 22.0 | 6388 | 53 | 75 | 2.5 |

**(b)**

| **Variety/**  **Seed type** | **Exposure** | **Exposure time (minutes)** | **Energy density (mJ cm^-2^)** | **Mean**  **Germination** | **% ± from control** | **S.E** |
| --- | --- | --- | --- | --- | --- | --- |
| FH-490, Bold seed | Control | Control | Control | 33 | - | 7.5 |
|  | E1 | 1.0 | 290 | 45 | 38 | 5.0 |
|  | E2 | 2.0 | 581 | 43 | 31 | 7.5 |
|  | E5 | 8.0 | 2323 | 55 | 69 | 5.0 |
|  | E6 | 10.0 | 2904 | 60 | 85 | 5.0 |
|  | E10 | 18.0 | 5227 | 50 | 54 | 0.0 |
|  | E12 | 22.0 | 6388 | 35 | 8 | 0.0 |
| FH-492, Bold seed | Control | Control | Control | 35 | - | 5.0 |
|  | E1 | 1.0 | 290 | 58 | 64 | 2.5 |
|  | E2 | 2.0 | 581 | 48 | 36 | 2.5 |
|  | E5 | 8.0 | 2323 | 50 | 43 | 5.0 |
|  | E6 | 10.0 | 2904 | 45 | 29 | 5.0 |
|  | E10 | 18.0 | 5227 | 65 | 86 | 5.0 |
|  | E12 | 22.0 | 6388 | 48 | 36 | 7.5 |

S.E = Standard Error
