# Supplementary material for: Enhancement of germination and yield of cotton through optical seed priming: Lab. and diverse environment studies
Source: PLoS One. 2023 Jul 20;18(7):e0288255. doi: 10.1371/journal.pone.0288255 (PMC10358893; doi:10.1371/journal.pone.0288255)
Supplement: S10 Table — (DOCX) [file pone.0288255.s010.docx]

**S10 Table (a, b). Germination (%) and percent increase in germination over control after seed irradiation with LED red light in controlled environment.**

**(a)**

| **Variety/**  **Seed type** | **Exposure** | **Exposure time (minutes)** | **Energy density (mJ cm^-2^)** | **Mean**  **Germination** | **% ± from control** | **S.E** |
| --- | --- | --- | --- | --- | --- | --- |
| Cyto-124, Bold seed | Control | Control | Control | 40 | - | 3 |
|  | Ea | 2.5 | 382 | 60 | 50 | 3 |
|  | E1 | 5.0 | 764 | 53 | 33 | 3 |
|  | E2 | 10.0 | 1528 | 47 | 17 | 3 |
|  | E3 | 14.0 | 2139 | 47 | 17 | 3 |
|  | E4 | 18.0 | 2750 | 60 | 50 | 5 |
| SADORI, Bold seed | Control | Control | Control | 45 | - | 5.0 |
|  | E3 | 4.0 | 795 | 58 | 28 | 2.5 |
|  | E5 | 8.0 | 1589 | 65 | 44 | 5.0 |
|  | E6 | 10.0 | 1987 | 65 | 44 | 0.0 |
|  | E9 | 16.0 | 3179 | 55 | 22 | 5.0 |
|  | E13 | 28.0 | 5563 | 65 | 44 | 0.0 |

**(b)**

| **Variety/**  **Seed type** | **Exposure** | **Exposure time (minutes)** | **Energy density (mJ cm^-2^)** | **Mean**  **Germination** | **% ± from control** | **S.E** |
| --- | --- | --- | --- | --- | --- | --- |
| SADORI, Fuzzy seed | Control | Control | Control | 25 | - | 5.0 |
|  | E3 | 4.0 | 795 | 35 | 40 | 5.0 |
|  | E5 | 8.0 | 1589 | 38 | 50 | 2.5 |
|  | E6 | 10.0 | 1987 | 45 | 80 | 0.0 |
|  | E9 | 16.0 | 3179 | 45 | 80 | 0.0 |
|  | E13 | 28.0 | 5563 | 33 | 30 | 2.5 |
| FH-492, Fuzzy seed | Control | Control | Control | 30 | - | 0.0 |
|  | E2 | 2.0 | 397 | 45 | 50 | 5.0 |
|  | E5 | 8.0 | 1589 | 45 | 50 | 5.0 |
|  | E6 | 10.0 | 1987 | 48 | 58 | 2.5 |
|  | E9 | 16.0 | 3179 | 45 | 50 | 5.0 |
|  | E13 | 28.0 | 5563 | 43 | 42 | 2.5 |

S.E = Standard Error
