# Supplementary material for: Enhancement of germination and yield of cotton through optical seed priming: Lab. and diverse environment studies
Source: PLoS One. 2023 Jul 20;18(7):e0288255. doi: 10.1371/journal.pone.0288255 (PMC10358893; doi:10.1371/journal.pone.0288255)
Supplement: S11 Table — (DOCX) [file pone.0288255.s011.docx]

**S11 Table (a, b). Germination (%) and percent increase in germination over control after seed irradiation with diode laser in controlled environment.**

**(a)**

| **Variety/**  **Seed type** | **Exposure** | **Exposure time (minutes)** | **Energy density (mJ cm^-2^)** | **Mean**  **Germination** | **% ± from control** | **S.E** |
| --- | --- | --- | --- | --- | --- | --- |
| Cyto-124, Bold seed | Control | Control | Control | 28 | - | 2.5 |
|  | E1 | 1.0 | 306 | 43 | 55 | 2.5 |
|  | E3 | 2.0 | 611 | 48 | 73 | 2.5 |
|  | E5 | 4.0 | 1223 | 30 | 9 | 0.0 |
|  | E6 | 5.0 | 1528 | 35 | 27 | 5.0 |
|  | E9 | 11.0 | 3362 | 33 | 18 | 2.5 |
| NIA-NOORI, Fuzzy seed | Control | Control | Control | 23 | - | 2.5 |
|  | E1 | 1.0 | 306 | 25 | 11 | 5.0 |
|  | E3 | 2.0 | 611 | 25 | 11 | 5.0 |
|  | E5 | 4.0 | 1223 | 30 | 33 | 5.0 |
|  | E6 | 5.0 | 1528 | 43 | 89 | 2.5 |
|  | E9 | 11.0 | 3362 | 45 | 100 | 5.0 |

**(b)**

| **Variety/**  **Seed type** | **Exposure** | **Exposure time (minutes)** | **Energy density (mJ cm^-2^)** | **Mean**  **Germination** | **% ± from control** | **S.E** |
| --- | --- | --- | --- | --- | --- | --- |
| SADORI, Bold seed | Control | Control | Control | 50 | - | 5.0 |
|  | E1 | 1.0 | 306 | 63 | 25 | 2.5 |
|  | E3 | 2.0 | 611 | 65 | 30 | 5.0 |
|  | E5 | 4.0 | 1223 | 75 | 50 | 5.0 |
|  | E6 | 5.0 | 1528 | 70 | 40 | 0.0 |
|  | E9 | 11.0 | 3362 | 63 | 25 | 2.5 |
| FH-490, Bold seed | Control | Control | Control | 33 | - | 7.5 |
|  | E1 | 1.0 | 306 | 40 | 23 | 5.0 |
|  | E3 | 2.0 | 611 | 43 | 31 | 2.5 |
|  | E5 | 4.0 | 1223 | 50 | 54 | 0.0 |
|  | E9 | 11.0 | 3362 | 43 | 31 | 2.5 |

S.E = Standard Error
