# Supplementary material for: Enhancement of germination and yield of cotton through optical seed priming: Lab. and diverse environment studies
Source: PLoS One. 2023 Jul 20;18(7):e0288255. doi: 10.1371/journal.pone.0288255 (PMC10358893; doi:10.1371/journal.pone.0288255)
Supplement: S12 Table — (DOCX) [file pone.0288255.s012.docx]

**S12 Table (a, b). Germination (%) and percent increase in germination over control after seed irradiation with UV-B light in controlled environment.**

**(a)**

| **Variety/**  **Seed type** | **Exposure** | **Exposure time (minutes)** | **Energy density (mJ cm^-2^)** | **Mean**  **Germination** | **% ± from control** | **S.E** |
| --- | --- | --- | --- | --- | --- | --- |
| Cyto-124, Bold seed | Control | Control | Control | 25 | - | 0.0 |
|  | E5 | 1.0 | 234 | 28 | 10 | 2.5 |
|  | E7 | 1.5 | 352 | 30 | 20 | 0.0 |
|  | E10 | 7.0 | 1641 | 35 | 40 | 0.0 |
|  | E14 | 15.0 | 3517 | 38 | 50 | 2.5 |
| NIA-NOORI, Fuzzy seed | Control | Control | Control | 25 | - | 5.0 |
|  | E5 | 60 | 234 | 30 | 20 | 5.0 |
|  | E7 | 1.5 | 352 | 45 | 80 | 5.0 |
|  | E10 | 7.0 | 1641 | 38 | 50 | 2.5 |
|  | E11 | 9.0 | 2110 | 38 | 50 | 2.5 |
|  | E14 | 15.0 | 3517 | 70 | 180 | 5.0 |

**(b)**

| **Variety/**  **Seed type** | **Exposure** | **Exposure time (minutes)** | **Energy density (mJ cm^-2^)** | **Mean**  **Germination** | **% ± from control** | **S.E** |
| --- | --- | --- | --- | --- | --- | --- |
| SADORI, Fuzzy seed | Control | Control | Control | 40 | - | 5.0 |
|  | E10 | 7.0 | 1641 | 43 | 6 | 2.5 |
|  | E14 | 15.0 | 3517 | 53 | 31 | 2.5 |
| FH-490, Bold seed | Control | Control | Control | 43 | - | 2.5 |
|  | E10 | 7.0 | 1641 | 45 | 6 | 5.0 |
|  | E14 | 15.0 | 3517 | 48 | 12 | 2.5 |

S.E = Standard Error
