# Supplementary material for: Enhancement of germination and yield of cotton through optical seed priming: Lab. and diverse environment studies
Source: PLoS One. 2023 Jul 20;18(7):e0288255. doi: 10.1371/journal.pone.0288255 (PMC10358893; doi:10.1371/journal.pone.0288255)
Supplement: S14 Table — Germination (%) and percent increase/decrease (±) in germination over control, and Cotton yield (kg ha-1) and percent ± in yield over control in field trial conducted at NIA, Tandojam after optical seed priming. (DOCX) [file pone.0288255.s014.docx]

**S14 Table. Environment 1 (Bold seed trial). Germination (%) and percent increase/decrease (±) in germination over control, and Cotton yield (kg ha^-1^) and percent ± in yield over control in field trial conducted at NIA, Tandojam after optical seed priming.**

| **S. No.** | **Variety/**  **Seed type** | **Treatment** | **Exposure** | **Exposure time (minutes)** | **Energy density (mJ cm^-2^)** | **Germination** | **% ± from control** | **Yield**  **(Kg ha^-1^)** | **% ± from control** |
| --- | --- | --- | --- | --- | --- | --- | --- | --- | --- |
|  | NIA Noori, Bold | Control | Control | - | - | 41 | - | 1098 | - |
|  | NIA Noori, Bold | Diode Laser | E6 | 5.0 | 1528 | 43 | 5 | 1711 | 56 |
|  | NIA Noori, Bold | UV-B | E14 | 15.0 | 3517 | 46 | 12 | 1915 | 74 |
|  | NIA Noori, Bold | UV-C | E6 | 1.5 | 132 | 36 | -12 | 1628 | 48 |
|  | NIA Noori, Bold | LED Blue | E12 | 22.0 | 6388 | 48 | 17 | 1915 | 74 |
|  | NIA Noori, Bold | LED Red | E2 | 2.0 | 397 | 36 | -12 | 1212 | 10 |
|  | Sadori, Bold | Control | Control | - | - | 43 | - | 1499 | - |
|  | Sadori, Bold | Diode Laser | E5 | 4.0 | 1223 | 53 | 23 | 1934 | 29 |
|  | Sadori, Bold | UV-B | E7 | 1.5 | 352 | 46 | 7 | 1610 | 7 |
|  | Sadori, Bold | UV-C | E6 | 1.5 | 132 | 49 | 14 | 1719 | 15 |
|  | Sadori, Bold | LED Blue | E12 | 22.0 | 6388 | 55 | 28 | 1647 | 10 |
|  | Sadori, Bold | LED Red | E6 | 10.0 | 1987 | 46 | 7 | 1730 | 15 |
|  | ANOVA | P value: |  |  |  | 0.2398 |  | 0.0001 |  |
|  | Coefficient of variation: | |  |  |  | 19.27 |  | 10.8 |  |
