# Supplementary material for: Enhancement of germination and yield of cotton through optical seed priming: Lab. and diverse environment studies
Source: PLoS One. 2023 Jul 20;18(7):e0288255. doi: 10.1371/journal.pone.0288255 (PMC10358893; doi:10.1371/journal.pone.0288255)
Supplement: S15 Table — Germination (%) and percent ± in germination over control and Cotton yield (kg ha-1) and percent ± in yield over control in field trial conducted at NIA, Tandojam after optical seed priming. (DOCX) [file pone.0288255.s015.docx]

**S15 Table. Environment 1 (Fuzzy seed trial). Germination (%) and percent ± in germination over control and Cotton yield (kg ha^-1^) and percent ± in yield over control in field trial conducted at NIA, Tandojam after optical seed priming.**

| **S. No.** | **Variety/**  **Seed type** | **Treatment** | **Exposure** | **Exposure time (minutes)** | **Energy density (mJ cm^-2^)** | **Germination** | **% ± from control** | **Yield**  **(Kg ha^-1^)** | **% ± from control** |
| --- | --- | --- | --- | --- | --- | --- | --- | --- | --- |
|  | NIA Noori, Fuzzy | Control | Control | - | - | 41 | 0 | 1286 | 0 |
|  | NIA Noori, Fuzzy | Diode Laser | E9 | 11.0 | 3362 | 52 | 27 | 1868 | 45 |
|  | NIA Noori, Fuzzy | UV-B | E14 | 15.0 | 3517 | 46 | 12 | 1144 | -11 |
|  | NIA Noori, Fuzzy | UV-C | E8 | 7.0 | 615 | 51 | 24 | 1304 | 1 |
|  | NIA Noori, Fuzzy | LED Blue | E12 | 22.0 | 6388 | 56 | 37 | 1684 | 31 |
|  | NIA Noori, Fuzzy | LED Red | E6 | 10.0 | 1987 | 48 | 17 | 1101 | -14 |
|  | Sadori, Fuzzy | Control | Control | - | - | 44 | 0 | 1363 | 0 |
|  | Sadori, Fuzzy | Diode Laser | E1 | 1.0 | 306 | 43 | -2 | 1480 | 9 |
|  | Sadori, Fuzzy | UV-B | E14 | 15.0 | 3517 | 48 | 9 | 1665 | 22 |
|  | Sadori, Fuzzy | UV-C | E8 | 7.0 | 615 | 49 | 11 | 2119 | 55 |
|  | Sadori, Fuzzy | LED Blue | E12 | 22.0 | 6388 | 45 | 2 | 1332 | -2 |
|  | Sadori, Fuzzy | LED Red | E6 | 10.0 | 1987 | 44 | 0 | 1175 | -14 |
|  | ANOVA | P value: |  |  |  | 0.3549 |  | 0.0000 |  |
|  | Coefficient of variation: | |  |  |  | 14.68 |  | 12.8 |  |
